# A vlincRNA participates in senescence maintenance by relieving H2AZ-mediated repression at the *INK4* locus

**DOI:** 10.1038/ncomms6971

**Published:** 2015-01-20

**Authors:** Sandra Lazorthes, Céline Vallot, Sébastien Briois, Marion Aguirrebengoa, Jean-Yves Thuret, Georges St. Laurent, Claire Rougeulle, Philipp Kapranov, Carl Mann, Didier Trouche, Estelle Nicolas

**Affiliations:** 1 Université de Toulouse; UPS; LBCMCP; F-31062 Toulouse, France; 2 CNRS; LBCMCP; F-31062 Toulouse, France; 3 UMR 7216, Université Paris Diderot, 75205 Paris, France; 4 CEA, iBiTec-S, SBIGeM/CNRS-FRE3377 and I2BC/Université Paris-Sud and Paris-Saclay, 91191 Gif-sur-Yvette, France; 5 St Laurent Institute, Woburn, Massachusetts 01801, USA; 6 Institute of Genomics, Huaqiao University School of Medicine, Xiamen 361021, China; 7 Academy of Biology and Biotechnology, South Federal University, Rostov-on-Don, Russia

## Abstract

Non-coding RNAs (ncRNAs) play major roles in proper chromatin organization and function. Senescence, a strong anti-proliferative process and a major anticancer barrier, is associated with dramatic chromatin reorganization in heterochromatin foci. Here we analyze strand-specific transcriptome changes during oncogene-induced human senescence. Strikingly, while differentially expressed RNAs are mostly repressed during senescence, ncRNAs belonging to the recently described vlincRNA (very long intergenic ncRNA) class are mainly activated. We show that *VAD*, a novel antisense vlincRNA strongly induced during senescence, is required for the maintenance of senescence features. *VAD* modulates chromatin structure in *cis* and activates gene expression in *trans* at the *INK4* locus, which encodes cell cycle inhibitors important for senescence-associated cell proliferation arrest. Importantly, *VAD* inhibits the incorporation of the repressive histone variant H2A.Z at *INK4* gene promoters in senescent cells. Our data underline the importance of vlincRNAs as sensors of cellular environment changes and as mediators of the correct transcriptional response.

## Abstract (web summary)

Senescence is associated with chromatin reorganization in heterochromatin foci. Here the authors show that *VAD*, a very long intergenic non-coding RNA activated by senescence, inhibits the incorporation of the repressive histone variant H2A.Z to *INK4* promoters in senescent cells.

## Graphical abstract

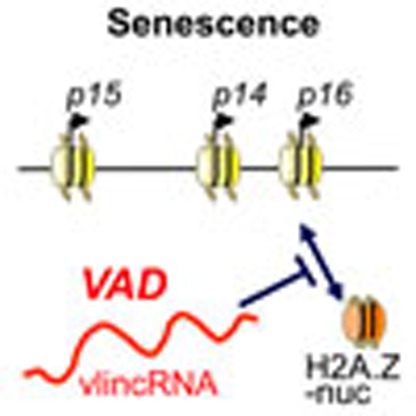

Senescence is a major anticancer barrier^1^,^2^,^3^ characterized by a permanent cell cycle arrest and triggered by telomere shortening or DNA damage, or by excessive mitogenic signals due to oncogene activation^4^. These signals activate the two major tumour suppressor pathways p16/Rb and p21/p53 (ref. 4), which are the two main pathways mediating senescence induction. The establishment of a specific genetic programme is another characteristic of cellular senescence including the expression changes in cell cycle regulators. Strikingly, senescent cells undergo major rearrangements of chromatin structure with the appearance of senescence-associated heterochromatin foci (SAHF) in the nucleus^5^,^6^,^7^. SAHFs are chromatin foci associated with heterochromatin marks and other chromatin proteins, such as the HMGA (High Mobility Group A) proteins, and are involved in the silencing of proliferation-related genes^5^,^6^,^7^. So far, analyses of the genome expression in senescence mostly focused on annotated protein-coding regions and microRNAs^8^,^9^, although a recent study described some expression changes of lncRNAs during replicative senescence^10^.

Non-coding RNAs (ncRNAs) are some of the major components required for proper chromatin function^11^. ncRNAs can be transcribed from known genes or from intergenic loci. Small, long (>200 nt, lncRNAs) and very long intergenic (>50 kb, vlincRNAs) ncRNAs are widespread in the human genome^12^,^13^,^14^,^15^. Their number now exceeds the number of protein-encoding mRNAs and understanding their function is still a challenge, especially in the case of very large RNAs (vlincRNA or macroRNA) whose unusual size leads to technical difficulties^16^. Antisense non-coding transcripts share complementarity with known RNAs, and mediate post-transcriptional regulation as well as transcriptional regulation through chromatin modifications of their corresponding mRNA^17^. Epigenetic regulation by long antisense RNA has been mostly studied in the contexts of genomic imprinting and during X chromosome inactivation. However, recent studies show their involvement in the transcriptional regulation of some non-imprinted autosomal loci^11^.

Formation of many heterochromatic regions, such as pericentric heterochromatin, involves ncRNAs^18^,^19^,^20^. ncRNAs could therefore be important for SAHF induction during senescence. However, little is known about the involvement of ncRNAs in the process of cellular senescence^9^. Here we provide the first analysis of strand-specific transcriptome changes in senescent versus proliferative cells, independent of gene annotation and at a high resolution, in particular allowing the characterization of unannotated ncRNAs such as novel antisense transcripts. This analysis allows us to identify novel RNAs belonging to the recently described class of very long (>50 kb) intergenic non-coding (vlinc) RNAs^14^,^15^, whose expression changes in senescence. We focus on a particular vlincRNA, *VAD* (Vlinc RNA Antisense to DDAH1), partially antisense to the *DDAH1* gene. *VAD* is produced from a single transcription unit of over 200 kb, is largely unspliced and weakly polyadenylated. We show its role in senescence maintenance and further characterize its molecular mechanisms of action in *cis* and in *trans* by regulating the expression of the *INK4* locus.

## Results

### Strand-specific expression changes in RAF-induced senescence

Senescence was induced in hTERT-immortalized WI38 human fibroblasts by oncogenic stress through hyperactivation of the ERK1/2 MAP kinases mediated by RAF1-ER fusion protein. On 4-hydroxy-tamoxifen (4-HT) addition, senescence entry is rapid and synchronous^21^. Proliferative WI38 hTERT RAF1-ER cells were cultured in physiological O_2_ levels (5%) to avoid oxidative stresses and premature senescence entry^21^. Senescence induction on 4-HT addition was very effective, as shown by the rapid and homogenous appearance of SAHF, the strong proliferation arrest and the increased expression of known senescence-induced markers such as the cyclin-dependent kinase inhibitors mRNAs and proteins (p21, p15 and p16) reflecting activation of the Rb and p53 pathways (Supplementary Fig. 1). We purified total RNAs from proliferative and senescent cells and interrogated them on tiling arrays covering human chromosomes 1 and 6. Using two different strategies for complementary DNA (cDNA) preparation, we were able to analyse RNAs transcribed from either strand of both chromosomes. We next developed an analysis procedure to identify all transcripts whose expression changed during senescence independently of the genomic annotations (Supplementary Fig. 2, Methods). Note that the first step of this analysis was based on the signal given by 12 consecutive probes and thus did not allow us to identify transcripts shorter than ~300 bp.

We found 1,141 transcribed regions (transfrags) that were differentially expressed in senescent cells (*P* <2.5 × 10^−2^ as calculated through data randomization; see Supplementary Data 1 for the list of differentially expressed transfrags). Of those, 2/3 were repressed (Table 1) likely due to the formation of transcription-deficient heterochromatin foci. The majority of the differentially expressed transfrags (1,049/1,141) overlapped partially or totally with at least one annotated gene (RefSeq database), some overlapping more than one gene. Among them, 911 differentially expressed transfrags were transcribed in the sense orientation relative to annotated genes and could correspond to pre-mRNAs and mRNAs. However, some of them did not exactly match known transcripts, since they contained non-coding regions adjacent to annotated genes or only parts of annotated genes, suggesting that they may be new ncRNAs (such as stand-alone functional intronic transcripts^22^). In addition, many differentially expressed transfrags contained regions that were antisense to known genes (353) or were totally intergenic transcripts (92) (Table 1), these intergenic and antisense transcripts (445/1141) being presumably non-coding. Although among these newly identified transcripts, a few could be non-characterized pre-mRNAs, our data suggest that >30% of all differentially expressed transcripts represented ncRNAs, underlining the complexity of genome expression during senescence induction.

### Antisense transcript regulation is widespread in senescence

We next assessed the extent of differentially expressed antisense transcription. For this purpose, we analysed the genes overlapping antisense differentially expressed transfrags. We found that differentially expressed antisense transcription was widespread during senescence, affecting 399 (11.2%) out of the 3,570 genes present on chromosomes 1 and 6 (Table 2, Supplementary Data 2; compared with 26.8% that harboured differentially expressed sense transcription). Among these 399 genes, 197 did not present any variations on the sense strand and 177 presented concordant expression changes for the sense strand transcript (transcripts from both strands being either repressed or activated on senescence; Supplementary Fig. 3a). Note that this latter antisense population might be overestimated because of spurious second strand synthesis during cDNA preparation^23^. However, we confirmed by strand-specific quantitative reverse transcription-PCR (qRT–PCR) the concordant increase in the sense and antisense transcription (*p21* AS) at two different locations within the gene encoding the p21/CDKN1A CDK inhibitor (Supplementary Fig. 3b). In such genes, antisense RNAs could favour the expression of the sense transcript^11^,^17^,^24^. Finally, only a subset (32/399) of the genes with differentially expressed antisense transcription presented opposite changes for sense and antisense transcription (Supplementary Fig. 3a), and could represent examples of antisense RNAs negatively regulating the expression of the sense mRNA^11^,^17^.

### Differentially expressed vlincRNAs during senescence

**Figure 1:**
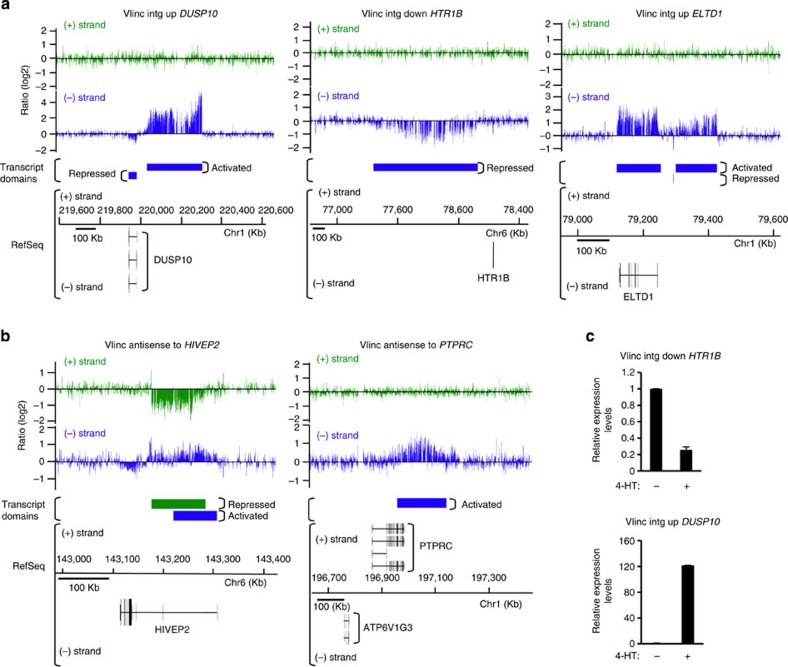
Examples of putative vlincRNAs and antisense vlincRNAs whose expression changes during senescence. (**a**) Schematic representations of the tiling array data (log2 of senescence/proliferative expression ratio, each bar corresponds to the ratio for one probe) for three putative differentially expressed vlincRNAs located in intergenic sequences. Transcript domains (differentially expressed transfrags) identified by the algorithm are indicated. The ratios and the domains are shown in green or in blue, for the (+) strand or for the (−) strand, respectively. Transcript variants from RefSeq, visualized in IGB, Affymetrix, are also indicated. Left panel: one activated vlincRNA transcribed from the (−) strand and upstream of the *DUSP10* gene, which is repressed. Middle panel: one repressed vlincRNA, transcribed from the (−) strand and downstream of the *HTR1B* gene. Right panel: one activated vlincRNA transcribed from the (−) strand and upstream of the *ELTD1* gene, which is activated. (**b**) Schematic representation of two differentially expressed antisense vlincRNAs, as in **a**. Left panel: one repressed vlincRNA ((+) strand) is antisense to the *HIVEP2* gene ((−) strand), which is partially activated during senescence. Right panel: one activated vlincRNA is transcribed from the (−) strand and is partially antisense to the *PTPRC* gene ((+) strand). (**c**) Validation of the expression changes in senescence of the differentially expressed vlincRNAs located downstream of the *HTR1B* gene and upstream of the *DUSP10* gene, by qRT–PCR (relative to *GAPDH* and normalized to 1 in proliferative cells). For the vlincRNA down *HTR1B*, the mean and s.d. from three independent experiments (*P* value<0.05, one-sided unpaired Student’s *t*-test) are shown. For the vlincRNA up *DUSP10*, the mean and s.d. from the qPCR sample triplicates of one representative experiment out of three are shown.

Interestingly, we observed differential expression for 71 very long transcripts (>50 kb) that did not match any annotated RNA (that is, outside of genes or in the antisense orientation to known genes) and thus correspond to the recently described vlincRNAs^14^,^15^ (Table 1, Supplementary Data 3). Examples of totally intergenic vlincRNAs either activated or repressed in senescence are shown in Fig. 1a and examples of differentially expressed vlincRNAs partially antisense to known genes are shown in Fig. 1b. Again, the expression changes were validated on selected differentially expressed vlincRNAs by qRT–PCR (Fig. 1c). Importantly, although vlincRNAs are known to be expressed in a highly tissue-specific fashion, 22 of these differentially expressed vlincRNAs were present among the 2,147 vlincRNAs recently identified in nine human cell lines^15^ (*P* value=7.44 × 10^−7^, calculated as in St. Laurent et *al*.^15^), further validating our vlincRNA analysis. We found that more than half (41/71, expected 23, *P* value=5 × 10^−6^, calculated with *χ*^2^test) of the differentially expressed vlincRNAs were induced during senescence in a striking contrast to overall differentially expressed transfrags for which about only one-third were activated, and the rest were repressed (Table 1). This unexpected statistical bias towards vlincRNA activation in senescence suggests that they are highly regulated and play an important role in senescence.

**Figure 2:**
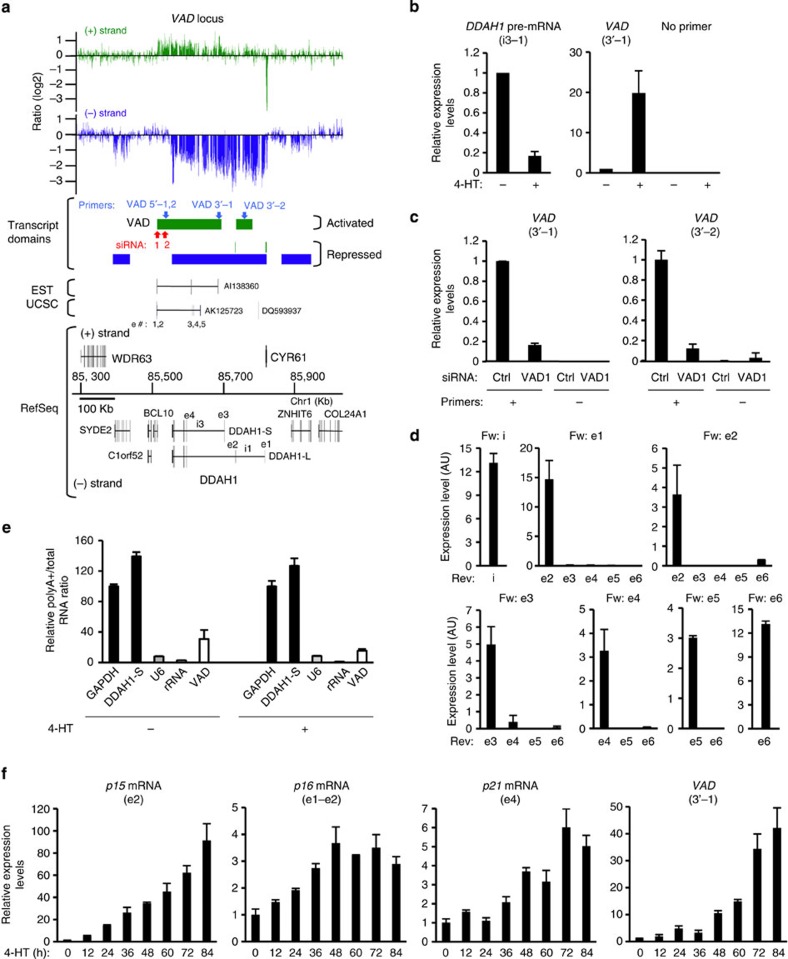
Characterization of the *VAD* antisense vlincRNA. (**a**) Schematic representation of the *VAD* locus with neighbouring genes, as in Fig. 1a. The UCSC gene AK125723 and the EST AI38360 that are co-linear to *VAD* are shown and their exons studied in **d** are labelled. Red and blue arrows, respectively, represent siRNAs and primers used for analysing *VAD*. Note that the *DDAH1* gene has two transcript variants that we called *DDAH1-S* for *DDAH1* small transcript variant (NM_012137.3) and *DDAH1-L* for *DDAH1* long transcript variant (NM_001134445.1). Exons and introns analysed by qPCR are indicated. Others genes from the locus are *SYDE2* (Synapse defective 1, Rho GTPase, homologue 2 (*C. elegans*)), *C1orf52* (Chromosome 1 open reading frame 52), *BCL10* (B-cell CLL/lymphoma 10), *CYR61* (cysteine-rich, angiogenic inducer, 61), *ZNHIT6* (Zinc finger, HIT-type containing 6). (**b**) Validation of the sense and antisense expression changes in senescence of the *DDAH1* pre-mRNA (using i3-1 primers) and of *VAD* (using VAD 3’-1 primers), by strand-specific qRT–PCR. The mean and s.d. from three independent experiments are shown, relative to *GAPDH* and normalized to 1 in proliferative cells. Note that the senescence-associated changes were always significant (*P* value<0.05, one-sided unpaired Student’s *t*-test) (**c**) Senescent WI38 hTERT RAF1-ER cells were transfected using the VAD1 siRNA (located at the 5′ end of *VAD*) and *VAD* expression was measured 72 h later by strand-specific qRT–PCR experiments using primers located at the 3′ end of the *VAD* RNA (see **a** for primer location). The mean and s.d. from three independent experiments is shown for VAD 3′-1 qRT–PCR (*P* value<0.05, one-sided unpaired Student’s *t*-test), and one representative experiment out of two is shown for VAD 3′-2 qRT–PCR (mean and s.d. from the qPCR sample triplicates), relative to *GAPDH* and normalized to 1 in siRNA control sample. (**d**) Total RNA from senescent cells was analysed by qRT–PCR using primers located in the indicated exons or intron (VAD 5′-1 primers) of the AK125723 RNA and the EST AI38360, as indicated. The e1/e2 PCR product also amplifies annotated *BCL10* mRNAs. Note that PCR products were amplified only using primers located in close proximity to each other in the genome (in the same exon or in intron). One representative experiment out of two is shown (mean and s.d. from the qPCR sample replicates). (**e**) Total or poly A+ RNA was prepared from WI38 hTERT RAF1-ER cells treated or not with 4-HT for 72 h, and analysed by qRT–PCR for the presence of *GAPDH* and *DDAH1-S* mRNAs, *U6* snRNP, ribosomal RNA and *VAD* (detected with VAD 5’-1 primers). The ratio between poly A+ and total RNA is indicated following normalization to 100 for GAPDH. One representative experiment out of two is shown (mean and s.d. from the qPCR sample triplicates). (**f**) WI38 hTERT RAF1-ER cells were induced to senescence, or not, for the indicated times. Total RNA was analysed for the expression of *p15*, *p16* and *p21* mRNA by qRT–PCR as well as *VAD* RNA by strand-specific qRT–PCR (with VAD-3′-1 primers). One representative experiment out of two is shown (mean and s.d. from the qPCR sample triplicates, relative to *GAPDH* and normalized to 1 in 0 h 4-HT).

Interestingly, a significant number of differentially expressed vlincRNAs (43, of which the majority (27) were activated in senescence) were antisense to known genes (Supplementary Data 3, see Figs 1b and 2a for examples). To gain insights into the function of these antisense vlincRNAs, we focused on a putative vlincRNA antisense to the *DDAH1* (dimethylarginine dimethylaminohydrolase 1) gene and strongly induced during senescence (Fig. 2a). Furthermore, the *DDAH1* gene was regulated in the opposite way, being strongly repressed during senescence. An interesting relationship of DDAH1 protein with senescence control can also be inferred from its known role in modulating nitric oxide levels, functionally linked to endothelial cell senescence^25^,^26^. In addition to *DDAH1*, tiling arrays indicate that three genes in the vicinity of the putative vlincRNA were also repressed (*SYDE2*, *CYR61*, *ZNHIT6*), whereas two were not affected (*BCL10* and *C1orf52*). Tiling array results for all these genes and for the putative vlincRNA (*VAD*) were confirmed by strand-specific qRT–PCR (pre-mRNA expression being measured in an intron^27^), qRT–PCR using random primers or western blots (Fig. 2b and Supplementary Fig. 4). Furthermore, this vlincRNA could also be detected in the ENCODE strand-specific long RNA-seq data^12^ from the nucleus of the human umbilical vein endothelial cell (HUVEC) cell line^15^ (Supplementary Fig. 5). Importantly, the increased expression of this putative vlincRNA was dependent on senescence induction and not observed in the parental cell line without the RAF1-ER fusion protein on 4-HT treatment (*VAD*, Supplementary Fig. 6).

### *VAD* possesses the characteristics of a vlincRNA

We first tested whether this putative differentially expressed vlincRNA corresponds to a single RNA molecule. We transfected senescent cells with two short interfering RNAs (siRNAs) against the 5′ end of the putative vlincRNA transcript domain (identified in our tiling array analysis). Both siRNAs decreased the level of RNA transcribed from the same strand, measured as much as >200 kb downstream from the siRNA target sequence (Fig. 2c and Supplementary Fig. 7). Thus, a unique RNA of at least 200 kb is produced at this locus. It does not correspond to any known protein-coding gene and is antisense to a known gene (*DDAH1*) making it an antisense vlincRNA (from now on referred to as *VAD* RNA, for VlincRNA Antisense to *DDAH1*).

UCSC Genes and EST databases have evidence, however, that spliced ncRNAs corresponding to the first 150 kb of the *VAD* RNA exist (Fig. 2a, transcripts AK125723 and AI38360). To test whether *VAD* is spliced, we performed qRT–PCR experiments using primer pairs located either in the same putative exons or connecting two putative consecutive exons per the spliced annotations above. We found that although the amount of *VAD* remained approximately constant throughout the 200 kb when analysed using primers at close proximity on the genomic DNA (in the same putative exons or in putative intron (Fig. 2d)), no amplification could be detected using primers from different putative exons (except for e1–e2), indicating that the splicing events described in AK125723 and AI38360 either did not exist or were very rare in our senescent cells. In addition, no evidence of efficient splicing within *VAD* locus could be observed through the analysis of strand-specific total RNA-seq data (Supplementary Fig. 8). Thus, these data strongly suggest that *VAD* is primarily an unspliced RNA molecule and presumably non-coding since it does not contain any ORF of over 100 amino acids from its 5′end, even though the possibility that some short peptides are produced from this region can not be excluded^28^.

In addition, *VAD* was less enriched than classical mRNAs after purification on a oligo-dT column (Fig. 2e), but more than the *U6* small nuclear RNAs or the 45S pre-ribosomal RNAs. This result, again confirmed by the high enrichment of *VAD* in HUVEC polyA-RNA as compared with HUVEC polyA+RNA in the ENCODE data^12^ (Supplementary Fig.5), indicates only partial polyadenylation, in agreement with described characteristics of vlincRNAs^14^,^15^. We next characterized the kinetics of *VAD* induction during senescence compared with the known senescence-associated genes^8^ encoding the p15, p16 and p21 cyclin-dependent kinase inhibitors. Expression of *p15* mRNA was very rapidly and strongly induced following RAF1 activation as compared to *p16* and *p21* mRNAs (Fig. 2f), presumably because it is a direct target of the MAP kinase pathways^29^. Interestingly, induction of *VAD* was also rapid and strong following RAF1 activation, in a striking parallel to *p15* mRNA activation. *VAD* could thus also be a direct target of oncogenic stress response pathways. In agreement with such a possibility, we found that it is also strongly induced following induction of senescence by activated MEK1 (ref. 30) (sixfold, similar to the induction of *VAD* by activated RAF1 (assessed by qRT-PCR using random primers, see Supplementary Fig. 4b)) in another human primary fibroblast cell line, IMR90, but not when WI38 or IMR90 cells undergo replicative senescence, and only weakly when senescence is induced on genotoxic stresses (Supplementary Fig. 9).

### *VAD* is required for the maintenance of senescent features

**Figure 3:**
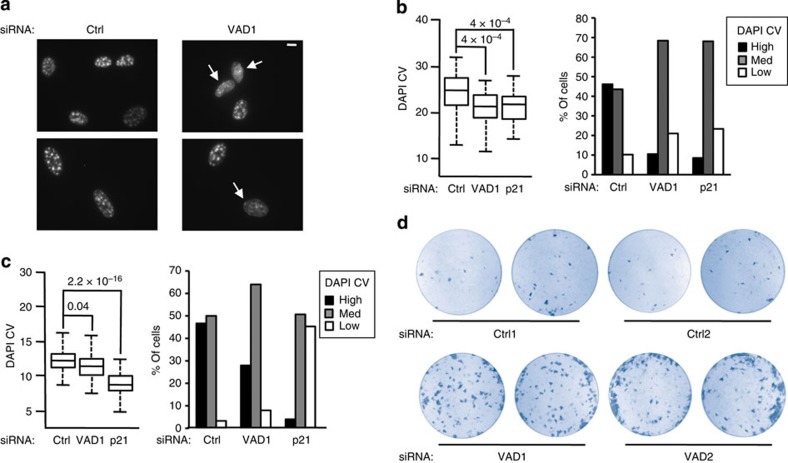
*VAD* expression is required for senescence maintenance. (**a**) Representative images of senescent WI38 hTERT RAF1-ER cells transfected using the indicated siRNA and stained with DAPI to visualize SAHF. Scale bar, 7 μm (**b**) Measurements of chromatin compaction in SAHF (DAPI CV) were performed on 50–100 cells. *P* values were calculated using Welch *t*-test assuming equal variance. One representative experiment out of three (performed using two different siRNAs) is shown. Right panel shows the distribution of cells according to their DAPI CV (high, med and low). Note that these classes were defined using all samples shown in Fig. 5e. (**c**) Same as in **b**, except that proliferative WI38 hTERT RAF1-ER cells were transfected then induced to senescence for 72 h. (**d**) Same as in **a**, except that cells were subjected to a clonogenic assay for 14 days before being stained with crystal violet. Two dishes are shown for each siRNA, from one representative experiment out of three.

Given that *VAD* expression is maximal when senescence is established, we tested its role in the maintenance of the senescent phenotype. Inhibition of *VAD* expression was achieved following transfection of senescent cells with two independent siRNAs (see Fig. 2c and Supplementary Fig. 7 for siRNA efficiency). Remarkably, *VAD* knockdown led to the appearance of cells without SAHF (see Fig. 3a for examples of transfected cells). Quantification of the heterogeneity of 4′,6-diamidino-2-phenylindole (DAPI) staining intensity (Fig. 3b), an indicator of SAHF compaction^31^, confirmed that SAHF maintenance was partially defective on *VAD* depletion. Strikingly, when comparing the effects of siRNA transfection before and after senescence induction, we noticed that *VAD* depletion affected more SAHF maintenance than SAHF formation (compare Fig. 3b,c), underlining the role of *VAD* in the maintenance of senescence features. SAHF are believed to be involved in the irreversible repression of proliferation-related genes^5^. In agreement with this idea, we found by clonogenic assays that some senescent cells transfected by VAD siRNAs were able to regain the ability to proliferate (Fig. 3d, see Supplementary Fig. 10 for validation of the assay using siRNAs against *p21* and *p16* mRNAs). Note that this effect is restricted to only a low proportion of cells, since we do not observe any increase in the number of BrdU-positive cells 72 h following *VAD* depletion (Supplementary Fig.11). Altogether, Fig. 3 results indicate that *VAD* depletion in senescent cells is sufficient, at least in part, to reverse some features of senescence.

### *VAD* regulates chromatin structure in *cis*

**Figure 4:**
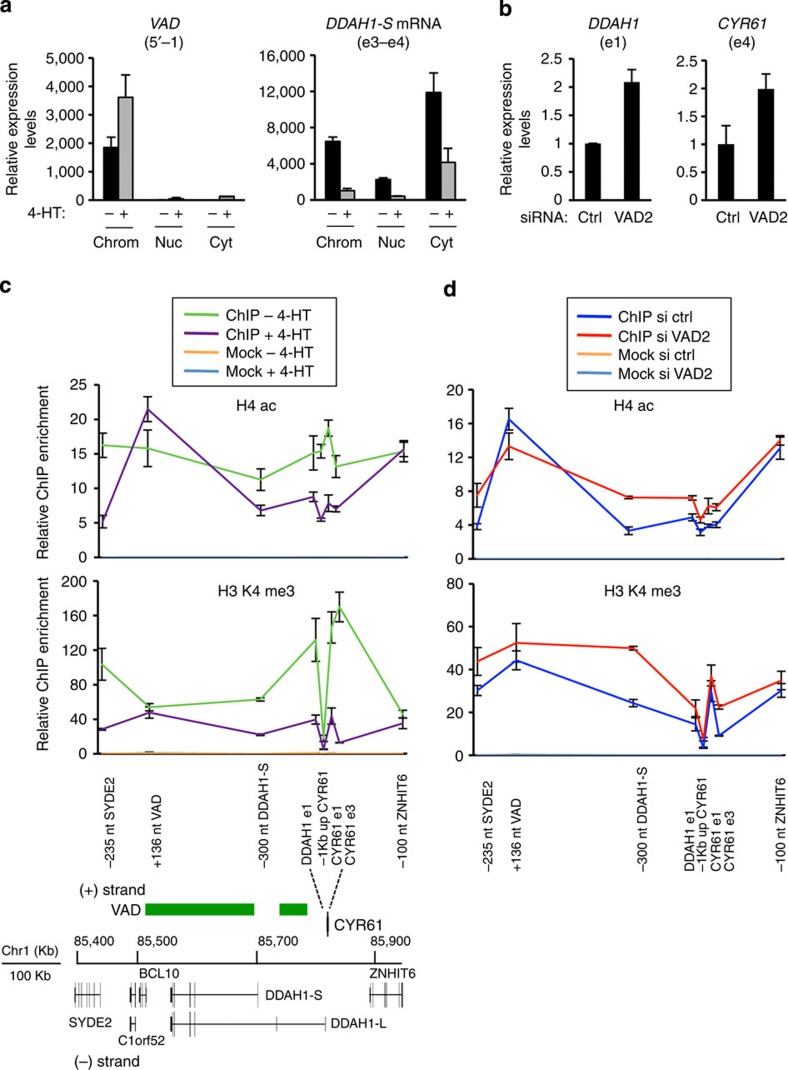
*VAD* regulates chromatin structure in *cis*. (**a**) WI38 hTERT RAF1-ER cells were induced to senescence for 72 h, or not, as indicated, and total RNA was then prepared from the cytoplasm (Cyt), the soluble nuclear fraction (Nuc) or the insoluble pellet (Chrom). The amount of the indicated RNA in the various fractions was analysed by qRT–PCR and calculated as described in the Methods. One representative experiment out of three is shown (mean and s.d. from the qPCR sample triplicates). *VAD* expression was assessed with VAD-5′-1 primers. (**b**) Senescent WI38 hTERT RAF1-ER cells were transfected by the indicated siRNA and subjected to run-on experiments analysed using the indicated primers. A representative experiment out of two is shown (mean and s.d. from the qPCR sample triplicates, relative to *GAPDH* and normalized to 1 in siRNA control). (**c**) WI38 hTERT RAF1-ER cells were induced to senescence for 72 h, or not, as indicated, then subjected to a ChIP assay using antibodies against acetylated histone H4 (H4ac), trimethylated lysine 4 of histone H3 (H3 K4me3) or no antibody (Mock) as a control. The amount of the indicated sequences was then quantified by qPCR and calculated relative to the input DNA. For each indicated sequence, the enrichment of the ChIP H4ac or H3 K4me3 was normalized to the enrichment of H3. The ratio between the indicated sequence enrichment and a control sequence (*U6*) was then performed. A schematic representation of the *VAD* locus with neighbouring genes (RefSeq hg18) is shown below the graphs. One representative experiment out of two or three is shown (mean and s.d. from the qPCR sample triplicates). (**d**) Senescent WI38 hTERT RAF1-ER cells were transfected by the indicated siRNA and subjected to a ChIP assay using a H3 K4me3 or H4ac antibodies. The indicated sequences were analyzed as in **c**. A representative experiment out of three is shown (mean and s.d. from the qPCR sample triplicates).

To gain insights into the molecular function of *VAD*, we investigated its subcellular localization. Cell fractionation experiments showed that *VAD* was almost exclusively found in the chromatin fraction both in proliferative and senescent cells (Fig. 4a), whereas, as expected, mRNAs, such as the *DDAH1-S* mRNAs, were predominantly cytoplasmic. This result suggests that *VAD* participates in the control of chromatin structure.

Strikingly, in tiling array experiments, we observed that many genes present in the vicinity of the *VAD* locus were repressed during senescence, including *DDAH1*, which is the antisense partner of *VAD* (see Fig. 2a and Supplementary Fig. 4 for validation). This is reminiscent of some genomic imprinting loci where long ncRNAs participate in the repression of many neighbouring genes by modifying chromatin structure in *cis*^32^,^33^,^34^. We thus tested whether *VAD* controls the expression of these neighbouring genes (*DDAH1*, *CYR61*, *SYDE2* or *ZNHIT6*). None of these mRNAs were consistently affected by *VAD* depletion (Supplementary Fig. 12), indicating that the repression of these genes occurring during senescence is mediated, at least in part, through *VAD*-independent mechanisms. In agreement with this possibility, kinetic analysis indicates that the repression of *DDAH1-L*, *DDAH1-S* and *CYR61* mRNAs did not strictly parallel *VAD* increase during senescence induction (compare Fig. 2f and Supplementary Fig. 13).

Nevertheless, by run-on experiments, we found that *VAD* depletion specifically increased the presence of nascent transcripts from the *CYR61* and *DDAH1* genes but not from the *SYDE2* gene (Fig. 4b and Supplementary Fig. 12) indicating that *VAD* can repress transcription of these genes. The lack of consistent effect of *VAD* depletion on their mRNA steady-state levels is probably due to strong *VAD*-independent post-transcriptional mechanisms.

Since *VAD* affects the transcription of *DDAH1* and *CYR61* genes, we next tested whether, as already shown for macroRNAs, *VAD* could regulate chromatin structure in *cis* on these two promoters. We first investigated the presence of chromatin marks in the vicinity of the locus during senescence induction. We found that H4 acetylation (H4ac) as well as H3 lysine 4 tri-methylation (H3K4me3), two chromatin marks linked to transcriptional activation, decreased during senescence on promoters or regions where transcription is repressed (*SYDE2* promoter, *DDAH1* promoters, *CYR61* promoter and coding region and the region between *DDAH1* and *CYR61* divergent promoters; Fig. 4c). In contrast, neither H4ac nor H3K4me3 changed on the promoter of the *ZNHIT6* gene, whose expression is only slightly repressed during senescence induction (Supplementary Fig. 4). On the *VAD* promoter, which is induced during senescence, H4ac increased but not H3K4me3 (Fig. 4c). Altogether, these data indicate that chromatin throughout the *VAD* locus experiences major changes during senescence induction.

Strikingly, in senescent cells transfected by *VAD* siRNAs, H4ac and H3K4me3 marks increased on all the regions where they would normally decrease in senescence (*SYDE2* promoter, *DDAH1* promoters, *CYR61* promoter and coding region and the region between *DDAH1* and *CYR61* divergent promoters; Fig. 4d), while remaining unchanged on the *ZNHIT6* or *VAD* promoters. Taken together, these data indicate that *VAD* expression is important for setting up the chromatin landscape around its locus in senescent cells by removing activating chromatin marks on repressed promoters.

### *VAD* functions in *trans* in the p16/Rb pathway

**Figure 5:**
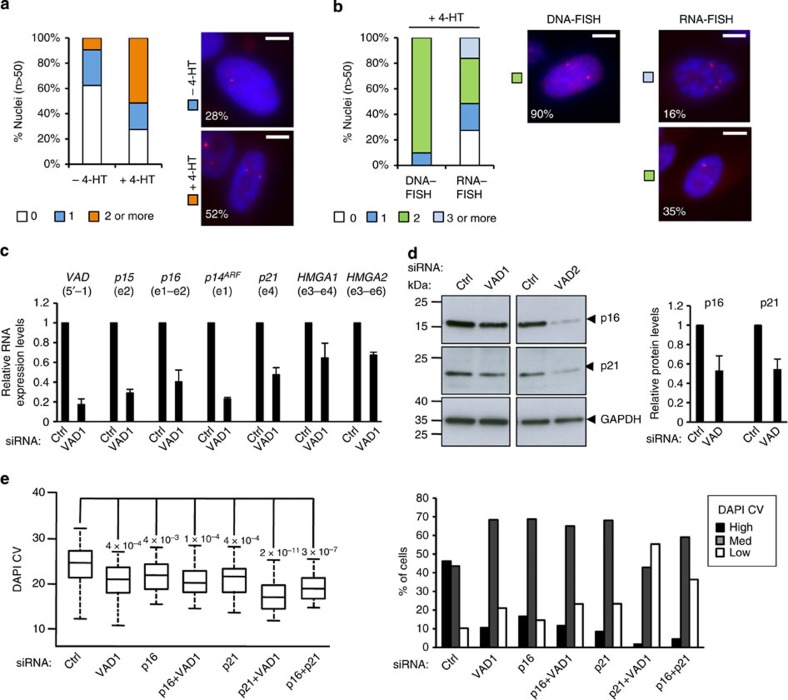
*VAD* functions in *trans* in the p15/p16 pathway. (**a**) *VAD* RNA-FISH experiments in WI38 hTERT RAF1-ER cells induced or not in senescence with 4-HT for 72 h. The number of pinpoints were counted in each cell for a population of *n*>50 cells. Representative images are shown with the indication of the percentage of cells with corresponding expression pattern. Scale bar, 5 μm. (**b**) *VAD* DNA- and RNA-FISH experiments in WI38 hTERT RAF1-ER cells induced in senescence with 4-HT for 72 h. The number of pinpoints were counted in each cell for a population of *n*>50 cells. Representative images are shown with the indication of the percentage of cells with corresponding expression pattern. Scale bar, 5 μm. (**c**) Senescent WI38 hTERT RAF1-ER cells were transfected using the indicated siRNA and the expression of the indicated RNAs was analysed by qRT–PCR 72 h later. The mean and s.d. from three independent experiments are shown, relative to *GAPDH* and normalized to 1 in siRNA control cells. Note that the effects of VAD siRNAs were always significant (*P* value<0.05, one-sided unpaired Student’s *t*-test). (**d**) Same as in **c** expect that whole-cell extracts were analysed by immunoblotting with the indicated antibodies. The right panel shows the quantification (using a G:Box camera) relative to GAPDH from three independent experiments (performed with two different siRNAs). Note that the effects of VAD siRNAs were significant (*P* value<0.05, one-sided unpaired Student’s *t*-test). (**e**) Senescent WI38 hTERT RAF1-ER cells transfected using the indicated siRNA and stained with DAPI. Quantification of DAPI CV and cell distribution were performed as in Fig. 3b. One representative experiment out of three is shown.

Our finding that *VAD* depletion does not induce consistent changes in the steady-state levels of the mRNAs in *cis* led us to address whether *VAD* could also act in *trans*. To test this possibility, we first investigated the expression and the localization pattern of *VAD* by FISH experiments. At the single cell level, we observed increased *VAD* staining in senescent cells, as expected: we could detect expression of *VAD* in 38% of proliferative cells and 73% of senescent cells (*P*=0.0003, Fisher's exact test). We also observed an increased number of cells with 2 or more dots in senescence, suggesting an increased biallelic expression in senescent cells (52 versus 9% in proliferative cells, *P*<0.0001; Fig. 5a). Interestingly, we observed some senescent cells (16%) with accumulation of *VAD* RNA into three dots or more, whereas such pattern was never observed in DNA-FISH where we detected one dot per allele in 90% of cells (Fig. 5b). These results suggest that *VAD* RNA could accumulate in senescent cells at other loci than its transcription sites, indicating the possibility of effects in *trans*.

To gain insights into the relevant targets of these *trans* effects, we investigated whether *VAD* regulates known senescence-linked pathways. Senescence is mainly controlled through two major pathways, the Rb and the p53 pathways. We first analysed the effect of *VAD* depletion on various components of these pathways induced during senescence, as well as on the expression of *HMGA1* and *HMGA2*, specifically involved in SAHF formation^6^ and greatly increased in RAF1-induced senescence (Supplementary Fig. 1).

Using a siRNA that induced a strong depletion of *VAD*, we found that *VAD* depletion in senescent cells affected the induction of all genes tested, including *p15* and *p16*, belonging to the Rb pathway, *p14*^*ARF*^ and *p21* from the p53 pathway as well as *HMGA1* and *HMGA2* (Fig. 5c). We confirmed that *VAD* depletion decreased the expression of p16 and of p21 proteins by immunoblot experiments (Fig. 5d). However, an siRNA, which depleted *VAD* less efficiently, significantly affected only *p15*, *p16* and *p14*^*ARF*^ mRNA expression (Supplementary Fig. 14), but not the expression of *HMGA1* and *HMGA2* or *p21* mRNA expression. We found the same results using a lower concentration of *VAD1* siRNA that decreased *VAD* less efficiently (Supplementary Fig. 14). These findings suggest that *p15*, *p16* and *p14*^*ARF*^, which are present in the same locus, the *INK4* locus (p16 and p14^ARF^ being alternative products of the *CDKN2A* gene and p15 encoded by the *CDKN2B* gene), could be the primary targets of *VAD.* On the other hand, *HMGA1* and *HMGA2* or *p21* could be secondary targets relying on *VAD* function in the regulation of the expression of *INK4* genes.

These data suggest that *VAD* would function in the Rb/p16 pathway rather than in the p53/p21 pathway. To confirm this hypothesis, we performed epistatic analyses by depleting *VAD* together with components of the Rb and p53 pathways (Fig. 5e). Indeed, depleting components from both the p53 and the Rb pathway together (p21 and p16) led to synergetic effects on SAHF maintenance. As expected for components of the Rb/p16 pathway, we found that *VAD* depletion synergized with depletion of p21 but not p16 in decreasing SAHF presence (Fig. 5e). Altogether, these data indicate that *VAD* favours senescence maintenance by promoting the expression of two components of the Rb pathway, *p15* and *p16*, in *trans*.

### *VAD* removes repressive H2A.Z from the *INK4* promoters

**Figure 6:**
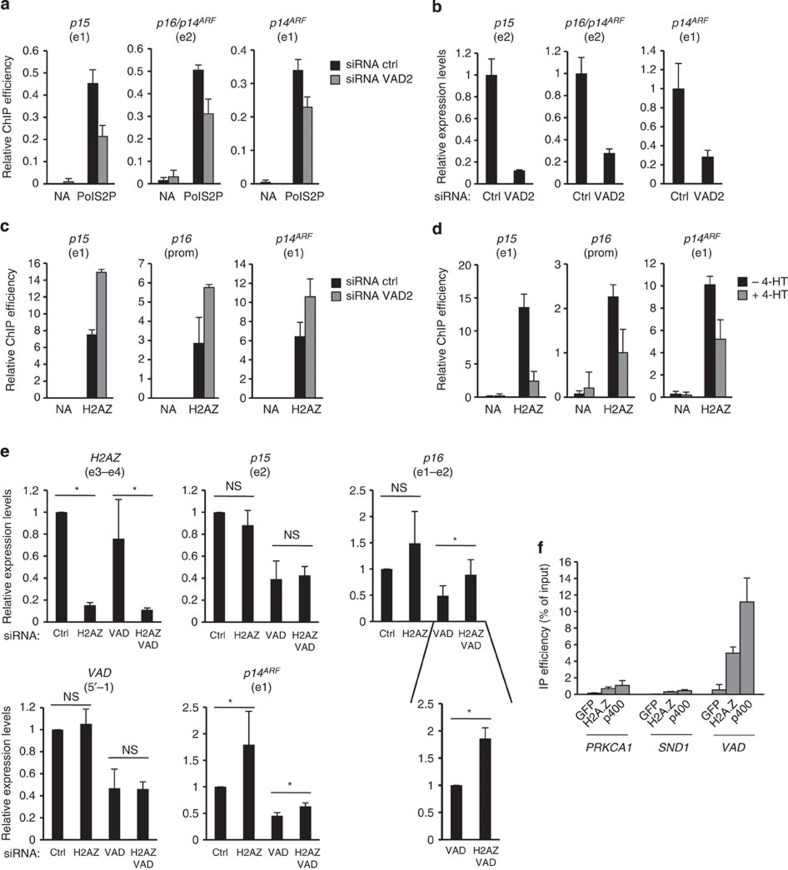
*VAD* prevents the H2A.Z-mediated repression of *INK4* promoters. (**a**) Senescent WI38 hTERT RAF1-ER cells were transfected by VAD siRNA and subjected to a RNA PolII phospho S2 (PolS2P) ChIP. The presence of *p15* and *p14*^*ARF*^ first exons and *p16*/*p14*^*ARF*^ second exon were tested by qPCR. A representative experiment out of two is shown (mean and s.d. from the qPCR sample triplicates). The amount of the indicated sequences was calculated relative to the input DNA and the enrichment for each indicated sequence was normalized to the enrichment of a control sequence (GAPDH exon 1). Note that given that all sequences transcribed to produce *p16* are also contained within *p14*^*ARF*^, we cannot assess the presence of RNA PolII phospho S2 on *p16* specifically. (**b**) Same as in **a**, except that a nuclear run-on was performed. A representative experiment out of two is shown (relative to *GAPDH* and normalized to 1 in siRNA control cells, mean and s.d. from the qPCR sample triplicates). (**c**) Same as in **a**, except that cells are subjected to a ChIP assay using antibodies against H2A.Z. The amount of the indicated sequences was then quantified by qPCR and calculated relative to the input DNA and as in Fig. 4c. A representative experiment out of two is shown (mean and s.d. from the qPCR sample triplicates). (**d**) Same as in **c**, except that WI38 hTERT RAF1-ER cells were induced to senescence for 72 h, or not, as indicated. One representative experiment out of two is shown (mean and s.d. from the qPCR sample triplicates). (**e**) Senescent WI38 hTERT RAF1-ER cells were transfected by VAD2 and H2AZ siRNAs as indicated and the expression of the indicated RNAs was analysed by qRT–PCR 72 h later. The mean and s.d. from three independent experiments are shown, relative to *GAPDH* and normalized to 1 in siRNA control cells. The insert shows the effect of H2A.Z siRNA on *p16* mRNA expression in *VAD*-depleted cells, standardized to 1 for cells transfected with VAD siRNA only. It shows that the effect of the H2A.Z siRNA is highly reproducible in this setting and that the large error bars in these samples in the whole panel reflect variations in the effects of *VAD* depletion from one experiment to the other. The star indicates significant effects of H2A.Z siRNAs (*P* value<0.05, one-sided unpaired Student’s *t*-test if control values were set to 1, one-sided paired Student’s *t*-test if not). NS, not significant. (**f**) H2A.Z- or p400-containing complexes were purified by immunoprecipitation from HeLa nuclear extracts and incubated with total RNA purified from senescent WI38 hTERT RAF1-ER cells. Bound RNA and input RNA were then analysed by qPCR for the presence of *VAD* or the indicated pre-mRNA of similar size. Bound RNA levels were calculated relative to the input RNA levels (% of input).

We next sought the mechanism by which *VAD* favours the expression of *INK4* genes in senescent cells. First, we found that *VAD* depletion in senescent cells affected the presence of elongating RNA Pol II at these genes, measured by chromatin immunoprecipitation (ChIP; Fig. 6a), indicating that *VAD* functions at the transcriptional level. This was directly confirmed by measuring nascent transcripts (Fig. 6b). Given that *VAD* is able to affect chromatin features, we next concentrated on chromatin marks. We found by ChIP experiments that chromatin marks known to correlate with transcriptional activation of the locus, such as H3K4me3 or H4ac, are not affected by *VAD* depletion, although they are induced, as expected, on senescence induction (Supplementary Fig. 15). We also tested the presence of the histone variant H2A.Z, since it can regulate the chromatin recruitment of Suz12, a member of the Polycomb PRC2 complex, involved in the repression of the *INK4* locus in proliferative cells^35^,^36^. We found that the presence of H2A.Z at the *INK4* promoters was strongly stimulated on *VAD* depletion in senescent cells (Fig. 6c). Moreover, H2A.Z association at the *INK4* promoters during senescence induction correlated with repression of these promoters, since its binding decreased during senescence progression (Fig. 6d), suggesting that it negatively regulates these promoters. We next tested the effect of H2A.Z depletion on the expression of *INK4* genes as well as on their decrease on *VAD* depletion. We did not find any change in p15 expression, perhaps because H2A.Z depletion was not efficient enough. However, we found that depleting H2A.Z led to an increase in *p14*^*ARF*^ expression (Fig. 6e). Moreover, depleting H2A.Z partly reversed the repression of *p16* expression by VAD siRNA. These results indicate that H2A.Z functions as a repressor at least for *p16* and *p14*^*ARF*^. Altogether, these data indicate that, during senescence induction, *VAD* decreases the presence of the repressive H2A.Z at *INK4* promoters, therefore promoting *p16* and *p14*^*ARF*^gene expression. Given that p16 is known to be a major mediator of senescence in this senescence system^21^, this certainly explains the role of *VAD* in senescence control.

To gain insights into the mechanism involved, we tested the possibility of a physical interaction between *VAD* and H2A.Z within its deposition machinery. RIP experiments were unsuccessful because chromatin extraction of *VAD* was very difficult and probably resulted in *VAD* cleavage due to its large size. As an alternate approach, we tested the interaction between *VAD* and H2A.Z-containing complexes *in vitro*. In such experiments, relevant protein/RNA physical interactions can be detected^37^. We thus immunoprecipitated native H2A.Z or p400-containing complexes (p400 being one of the human proteins known to mediate H2A.Z incorporation in chromatin^38^) to purify complexes involved in H2A.Z deposition, and we incubated them with RNAs purified from senescent cells. We next recovered RNAs bound to H2A.Z-containing complexes, and analysed for the presence of specific RNAs by qRT–PCR. We found that *VAD* could specifically interact with both H2A.Z and p400-containing complexes, whereas other RNAs of the same size were not enriched (Fig. 6f). Thus, these data indicate that *VAD* can specifically associate with H2A.Z and its deposition machinery, suggesting that *VAD* decreases H2A.Z occupancy at the *INK4* promoters through direct mechanisms.

## Discussion

Here we present analysis of the strand-specific transcriptome changes during oncogene-induced senescence. To our knowledge, this is the first such analysis and it shows widespread transcriptional changes during this process, affecting more than 25% of annotated genes on chromosomes 1 and 6 during senescence. Strikingly, most of these transcripts (67%) are repressed during senescence. Such level of repression is expected, since senescent cells harbour repressive heterochromatin foci and since all cell cycle-dependent genes are likely repressed in non-proliferating senescent cells. However, one-third of regulated genes are induced during senescence, indicating that this process is accompanied by the setting up of a specific genetic programme. This result reinforces the current notion that induction of senescence presents the characteristics of terminal differentiation. Whether this genetic programme is observed in all types of senescence, including replicative senescence, awaits future investigation.

We also observed a widespread modification of the expression of antisense transcription, with ~10% of the genes on chromosomes 1 and 6 seeing their antisense transcription changing during senescence. Although this could be an indirect consequence of major chromatin remodelling associated with senescence, such antisense transcripts could also directly participate in gene regulation. In agreement with this hypothesis, similar or opposite expression changes of these antisense transcripts and their corresponding sense transcripts are common during senescence.

Finally, we observed the presence of very large previously uncharacterized RNAs. Although some of them could be new pre-mRNAs, most of them are likely to belong to the recently described vlincRNA family, which consists of very long RNA largely unspliced and non-polyadenylated. Strikingly, in contrast to all the others classes of transcripts that we observed in our analysis, transcription of these vlincRNAs mainly increases during senescence, strongly suggesting that they play a role in senescence control. So far, only a handful of such RNAs with similar characteristics, macroRNAs, have been recognized and implicated in the control of imprinting and X chromosome inactivation^32^,^33^. We show that their number and function could be far greater and common than anticipated based on the number of known macroRNAs. Indeed, if the density of vlincRNAs is equivalent on all chromosomes, our data suggest that for the whole genome, the expression of more than 400 vlincRNAs changes during senescence. Strikingly, this number is not very different to the total number of vlincRNAs identified in immortalized cells by St Laurent et *al*.^15^ (about 600), suggesting that vlincRNA expression is highly regulated during senescence induction.

We next investigated the function of one such vlincRNA in senescence control by siRNA-mediated depletion. We found that two independent siRNAs directed against *VAD* inhibit maintenance of some senescence features, ruling out off-target effects. However, given that these two siRNAs both target the 5′ region of *VAD*, we cannot rule out the possibility that smaller RNAs targeted by these siRNAs and produced at the same locus mediate this control of senescence. Nevertheless, we believe that the effects we observed are indeed due to *VAD* inhibition, since we did not find any evidence for high levels of smaller RNAs produced from this locus by strand-specific RNA-seq experiments (Supplementary Fig. 8).

Using siRNA-mediated depletion, we found that *VAD* depletion affects chromatin structure in *cis*, by favouring the presence of positive chromatin marks at the promoters of neighbouring genes. This mechanism of chromatin control in *cis* by *VAD* is reminiscent of the function of the *Air* non-coding macroRNA in the silencing of three imprinted genes at the *Igf2r* cluster^34^. Thus, our work suggests that the regulation of chromatin structure over a multi-gene locus in *cis* by vlincRNAs or macroRNAs is probably much more common than previously anticipated and not restricted to genomic imprinting. The mechanism by which *VAD* functions in *cis* is unclear. However, we found by run-on experiments that transcription of the *DDAH1* and *CYR61* genes are increased on *VAD* depletion, although no consistent increase could be seen when analysing steady-state mRNA levels, perhaps because *VAD* depletion by siRNA was not effective enough. However, these data suggest that, beside the regulation of transcription and of chromatin marks by *VAD* in *cis*, the expression of those mRNA is likely regulated by strong *VAD*-independent post-transcriptional mechanisms. Thus, in *cis, VAD* mainly acts through chromatin regulation, which could participate in the global changes in chromatin structure associated with senescence.

**Figure 7:**
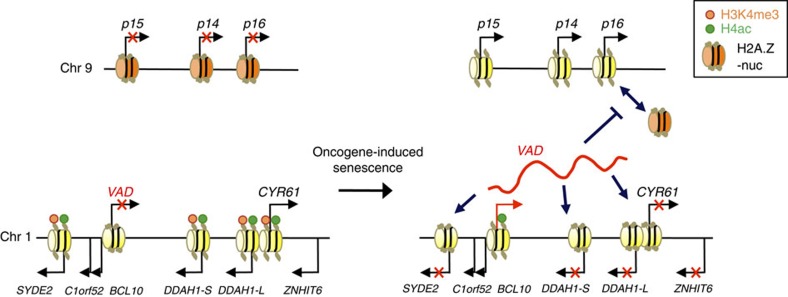
Model of the *VAD* mechanisms of action in senescence. The expression of the *VAD* vlincRNA is activated on oncogene-induced senescence. *VAD* regulates chromatin in *cis* by promoting the decrease of transcription-linked marks H4ac and H3K4me3 and in *trans* at the *INK4* locus by promoting the removal of repressive H2A.Z, which favours the expression of the *INK4* locus genes.

However, our data indicate that *VAD* function in the maintenance of some senescence features is most likely through its *trans* activating effect at the *INK4* locus, which is a master regulator of senescence. We found that *VAD* inhibits the occupancy of H2A.Z at *INK4*-encoding genes promoters. Importantly, this effect of *VAD* is not an indirect consequence of senescence inhibition. Indeed, whereas H3K4me3 or H4ac levels change at *INK4* promoters during senescence progression, *VAD* depletion does not reverse these changes (Supplementary Fig. 15), indicating that *VAD* depletion does not reverse all senescence-associated chromatin marks at these promoters. Thus, *VAD* specifically controls the presence of H2A.Z at *INK4* promoters. In addition, we found that *VAD* has the ability to specifically interact with the H2A.Z deposition machinery. We propose that through this physical interaction, *VAD* controls the deposition of H2A.Z, at least at some specific sites (see our model on Fig. 7). Altogether, these data suggest that *VAD* directly controls the occupancy of H2A.Z at *INK4* promoters.

A possible mechanism could be that *VAD* sequesters H2A.Z and its deposition machinery. The fact that H2A.Z or p400 depletion is sufficient to induce senescence^39^,^40^ indeed suggests that sequestering the H2A.Z deposition machinery could be an efficient way to maintain senescence. However, how such sequestration mechanisms could lead to a specific decrease of H2A.Z at some promoters and not elsewhere in the genome is unclear. One possibility could be that *VAD* interacts with a subset of H2A.Z-containing complexes, which are specifically involved in H2A.Z deposition at the *INK4* locus. An alternative possibility could be that *VAD* is recruited in the vicinity of the *INK4* locus to mediate its effect locally. The fact that *VAD* can localize outside its transcription sites in senescent cells is consistent with such a possibility, which requires further investigation.

Importantly, through the regulation of H2A.Z occupancy, *VAD* controls the expression of *INK4*-encoding genes. Indeed, we demonstrate for the first time that H2A.Z functions as a repressor of *INK4*-encoding genes. Such a finding probably explains why p400, a H2A.Z incorporating enzyme, can inhibit senescence induction through the p16 pathway^40^. Thus, our data allow us to propose that *VAD* favours senescence maintenance by blocking the incorporation of H2A.Z at the *INK4* promoters to maintain tumour suppressor gene expression. Understanding the mechanism by which H2A.Z represses *INK4* promoters will require further investigation. However, we can speculate on the importance of the Polycomb complex in this mechanism. Indeed, H2A.Z and the Polycomb protein Suz12 control the same set of genes in mouse ES cells, and the presence of H2A.Z is required for Suz12 recruitment in these cells^36^.

This work shows the importance of very long intergenic ncRNAs in the maintenance of some senescence features. So far, the only description of such a role for ncRNAs was restricted to microRNAs, implicated in transcriptional gene silencing at E2F-target promoters during RAS^V12^-induced senescence^9^. We now demonstrate the importance of a vlincRNA for the maintenance of senescence features (including proliferation arrest and SAHF) and chromatin regulation of a whole locus in *cis* and in *trans* (Fig. 7). If such a mechanism is common, many other vlincRNAs important for senescence maintenance likely exist. Strikingly, we found that some senescent cells transfected by siRNAs decreasing *VAD* expression can regain the ability to proliferate. Thus, our data indicate that it is possible to revert the senescent phenotype, including the permanent cell cycle arrest, by targeting ncRNAs, a property described so far for only a handful of proteins and for the microRNA *let-7f* (refs 6, 9, 21, 41, 42, 43).

Finally, LTR-promoter driven vlincRNA activation has been linked to cancer and the pluripotent state^15^. While the function of *VAD* is important for permanent cell cycle arrest, and more generally a global activation of vlincRNAs during senescence, is apparently not consistent with a cancer or stem cell phenotype, such a finding is reminiscent of the HMGA proteins. Indeed, HMGA proteins are important for the maintenance of the senescence phenotype, including the associated permanent cell cycle arrest, but are heavily over expressed in many human cancers^44^. Along the same line, the fact that *VAD* is expressed very early following oncogene-activation indicates that it is a target of pro-proliferative pathways. Interestingly, we found that induction of *VAD* is also observed in another cell line following oncogene-induced senescence but not on replicative senescence (Supplementary Fig. 9). Thus, one could envision that the physiological role of *VAD* is to further restrict the proliferative ability of senescent precancerous cells to ensure that they would not escape senescence and become malignant. More generally, our data suggest that modifications of vlincRNA expression could participate in the genome reprogramming associated with cell transformation or with trans-differentiation processes.

## Methods

### Cell culture

WI38-derived cells^21^, IMR90 (purchased at the ATCC) and IMR90 hTERT MEK1-ER cell line^30^ were maintained in MEM supplemented with glutamine, non-essential amino acids, sodium pyruvate, penicillin–streptomycin and 10% fetal bovine serum as per the ATCC in normoxic (5% O_2_) culture conditions. For senescence induction, cells were treated with 20 nM 4-HT (Sigma). siRNA transfection was performed using the Dharmafect 4 reagent (Dharmacon) according to the manufacturer’s recommendations, except that 100 nM of siRNA was used. For senescence maintenance assays, cells were treated 72 h with 4-HT, then transfected and cultured without 4-HT (for clonogenic and SAHF presence assays) or cultured without 4-HT for 24 h then reinduced 48 h with 4-HT (when monitoring RNA expression). For clonogenic assays, cells were seeded in 24 well plates at a density of 50,000 cells per well, induced in senescence for 3 days and then transfected with siRNA as described above for 3 days. One-third of cells from each well was seeded in new 6-cm plates and cultured for 10 to 14 days before staining with PBS+0.1% crystal violet.

### RNA extraction and reverse transcription

Total RNA or poly A+RNA were prepared using the MasterPure RNA Purification Kit (Epicentre Biotechnologies) supplemented with Baseline-ZERO DNAse (Epicentre) or the Oligotex direct mRNA minikit (Qiagen), respectively, according to the manufacturers’ recommendation. RNA (200 ng) was used for each reverse transcription experiment. Strand-specific reverse transcriptions were performed at 55 °C using the Sensiscript and Omniscript enzymes (Qiagen) according to the manufacturer’s recommendations. Each strand-specific reverse transcription was performed with one specific primer as indicated in the figures. In each experiment, we included a reverse transcription reaction without primer to monitor for DNA contamination. Non-strand specific reverse transcriptions were performed using random hexamers and Superscript III (Invitrogen) at 50 °C according to the manufacturer’s recommendations. In each experiment, we included a control without the reverse transcriptase to monitor for DNA contamination.

For microarray experiments, to determine transcription of both DNA strands, two different cDNAs populations were produced from the total RNA. cDNA1 was synthesized from 250 ng of total RNA (without rRNA reduction) according to the ‘GeneChip Whole Transcript Sense Target Labeling Assay’ (Affymetrix) and is identical to RNA. cDNA2 was synthesized from 12 μg of total RNA, as previously, but starting from the second cycle of cDNA synthesis thus skipping the first cycle cDNA synthesis and the ‘*In Vitro* Transcription’ amplification step. Therefore, cDNA2 is complementary to RNA. Differential cDNA populations were hybridized on the GeneChip Human Tiling 2.0R-A arrays (Affymetrix), which contain DNA probes from the forward DNA strand. Hybridization and scanning were performed at the Plateforme Biopuces (INSA, Toulouse). Plus and minus strand expression were monitored by the cDNA2 and cDNA1 experiments, respectively.

### Tiling array data analysis

Scanned array data normalization (quantile normalization, scale to 500) as well as calculation of the log2 (ratio) of the senescence signal over the control signal were performed using TAS (Affymetrix). For images, data were calculated using a bandwidth of 300 and visualized using the Integrated Genome Browser (IGB, Affymetrix) genome version H_sapiens_Mar_2006 with RefSeq (NetAffx (QuickLoad)).

For analysis, we worked on tiling array ratios (senescence signal over control signal) derived from two independent experiments. We developed an algorithm whose first step aims to analyse independently the two independent experiments, which means finding domains over each of them. The programme screens each data set along the chromosomes using a sliding window of 12 probes. When 10 probes in the window are above or below +0.35 or −0.35 for activation or repression respectively, a domain begins, and it spreads as long as the following window also satisfies it. We next used a new sliding window (50 probes) that goes on both sides of each domain to expand them as long as the window’s average stays above/below (activated/repressed) 0. When expanding the domains, some of them overlap and they are thus merged. At this step, the algorithm returns a set of domains (putative transcripts whose expression changes during senescence) for each experiment.

The second algorithm step aims to analyse the two experiments together. First, the algorithm eliminates transcripts that are found in only one experiment. Second, we made a virtual experiment, which is an average of the two actual experiments and which is used for the next algorithm steps. We observed that the transcripts have to be refined in term of boundaries. To specify the boundaries, at each side of a domain (that is, the first and last probes), the algorithm either reduces or expands the domain, probe after probe. For each considered probe, the algorithm considers 15 probes downstream and 15 probes upstream and averages the signal over this 30 probes window. When looking at activation, if the window’s average is greater than half the domain’s average, the domain expands, otherwise, it reduces. When looking at repression, the domain expands if the window’s average is lower than half the domain’s average and reduces otherwise. Finally, if some domains are overlapping, they get merged. We end up with a final set of domains, which is shown in Supplementary Data 1. Each domain corresponds to a transfrag whose expression changed during senescence. Through comparison of genome annotations (RefSeq from UCSC, Human genome assembly March 2006 (NCBI36/hg18), we obtained a list of genes that overlap the domains (Supplementary Data 1).

### Run-on experiments

Run-on experiments were performed using the Click-iT Nascent RNA Capture Kit (Molecular Probes, Life Technologies) according to the manufacturer’s recommendations. Cells were incubated for 2 h with 0.2 mM of 5-ethynyl uridine before harvesting.

### DNA- and RNA-FISH

Cells were grown on coverslips, permeabilized for 5 min on ice with CSK buffer (100 mM NaCl, 300 mM sucrose, 3 mM MgCl_2_, 10 mM Pipes, adjusted to pH 6.8 with NaOH and freshly supplemented with 2 mM Vanayl Ribonucleodide Complex (VRC, Biolabs) and with 1 mM EGTA), and fixed for 10 min in PBS supplemented with 3% paraformaldehyde and 2 mM VRC. After three washes with ice-cold ethanol 70%, coverslips were stored in ethanol 70% at −20 °C. Alexa594-labelled probes were generated by nick translation for *VAD* (fosmid G248P88891A3). Probes were precipitated with human Cot-1 DNA (Life Technologies), resuspended in formamide and hybridization buffer, denatured for 7 min at 75 °C and additionally preincubated 15 min at 37 °C. For DNA-FISH, coverslips were incubated for 1 h with RNaseA (100 μg μl^−1^, Life Technologies) and denatured 15 min at 80 °C in 70% Formamide/saline sodium citrate (SSC, 2 × ). Coverslips were then dehydrated in 70, 90 and 100% ethanol and incubated overnight with probe at 37 °C. For RNA-FISH, coverslips were dehydrated in 90 and 100% ethanol and incubated overnight with probe at 37 °C. For all RNA- and DNA-FISH, after three 50% formaldehyde/SSC (2 × ) washes and three SSC (2 × ) washes at 42 °C for 4 min, coverslips were mounted in Vectashield plus DAPI.

All images were taken on a fluorescence microscope Axioplan 2 Imaging (Zeiss) with a cooled Coolsnap camera (Roper Scientifics) controlled by the Metamorph 7.04 software (Roper Scientific) using a Plan-neofluar × 100 oil objective (numerical aperture 1.3, Zeiss). Optical Z-sections were collected at 0.2-μm steps through each nucleus at different wavelengths depending on the probes used (DAPI (360 nm, 470 nm) and Texas Red (596 nm, 612 nm)); ~25 optical sections per nucleus were collected. Stacks were processed using ImageJ 1.46, and three-dimensional FISH experiments are represented as a two-dimensional projection of the stacks (maximum projection).

### BrdU staining

Cells were seeded in Labtek plates at a density of 50,000 cells per well for proliferating cells and 100,000 cells per well for senescent cells. BrdU was added to media at the final concentration of 50 μM for 24 h. Cells were then washed with PBS supplemented with 1% bovine serum albumin (BSA), then fixed for 15 min at room temperature (RT) with 3.7% formaldehyde. Following three washing steps in PBS+ 1% BSA, cells were permeabilized by incubating them in PBS supplemented with 0.4% Triton-X-100 for 5 min at RT with agitation, and stained with DAPI in PBS for 5 min. Cells were washed and the fixation and permeabilization steps were repeated once. Cells were then treated with HCl 4 N for 10 min at RT with agitation, then washed three times using PBS+ 1% BSA. Following a saturation step (10 min with PBS+ 1% BSA), cells were incubated with the anti-BrdU antibody (sc-32323, 1/100 dilution) in PBS+ 1% BSA for 1.5 h at RT with agitation. Cells were washed three times with PBS+ 1% BSA and incubated for 1 h with the secondary anti mouse antibody conjugated to Alexa 594 (1/500 dilution). Cells were then washed three times (PBS+ 1% BSA) and mounted in Vectashield (Vector Laboratories Inc.).

### DAPI staining

Cells were fixed (in PBS+ 3.7% paraformaldehyde) for 15 min, washed 3 times in PBS, permeabilized in ethanol 70% for 15 min and stained with Vectashield with DAPI (Vector Laboratories Inc.). For DAPI CV measurement, 50 to 100 cells were analysed for DAPI heterogeneity using an ImageJ plugin^31^. The compaction of DNA in chromatin and the resulting striking inhomogeneity in two-dimensional DAPI pictures obtained by high-resolution fluorescent microscopy, resulted in an increase in the coefficient of variation (CV, s.d. as a percentage of the mean) of fluorescence intensity in the nucleus^31^. Subsequent statistical analysis was carried out with R. For each experiment, all analysed cells were ranked according to their DAPI CV and separated into three equal categories (High, Medium and Low). The distribution of the cells in these categories was then calculated for each siRNA.

### qPCR and western blots

qPCR analysis was performed on a CFX96 Real-time system device (BioRad Laboratories) using the IQ SYBR Supermix (BioRad), according to the manufacturer’s instructions. All samples were analysed in triplicates. Representative experiments show the mean and s.d. from these triplicates. For RNA expression analyses, all data were normalized relative to *GAPDH* mRNA levels (except for the cell fractionation (Fig. 4a), polyA+RNA (Fig. 2e) and exon–exon qPCR amplification (Fig. 2d) experiments). For the cell fractionation experiments, the qPCR quantification was multiply by the ratio of the cell fraction total RNA amount over the total RNA amount of the three fractions. Wole cell protein extracts were prepared in 1% SDS, 1 mM sodium vanadate, 10 mM Tris pH 7.4, 1% Triton, 0.5 M NaCl, supplemented with protease inbibitors (Roche) and phosphatase inhibitors (Sigma) with sonication until the viscosity of the sample is reduced. Western blots were performed using standard procedures (primary antibody dilutions to 1/1,000 except for GAPDH antibody that was diluted 1/10,000, full-length scans of western blot from Fig. 5d are provided in Supplementary Fig. 16).

### Chromatin immunoprecipitations

Cells (15 × 10^6^; induced or not in senescence, or induced in senescence and treated with siRNA) were crosslinked using 1% formaldehyde directly in the culture medium for 15 min. Then, 0.125 M Glycine was added for 5 min. After two washes with PBS, cells were collected and frozen at −80 °C. Cells were lysed with 3 ml of the following buffer: Pipes (5 mM, pH 8), KCl (85 mM), NP40 (0.5%) and homogenized with a dounce. All the buffers for the ChIP experiment were supplemented with EDTA-free protease inhibitor cocktail (Roche). Nuclei were harvested after centrifugation and sonicated 10 times for 10 s (power setting 5 and 50% duty cycle, Branson Sonifier 250) in 1.5 ml nulear lysis buffer (50 mM Tris pH 8.1, 10 mM EDTA, 1% SDS), to obtain DNA fragments of about 500 bp. DNA concentration was determined using a Nanodrop and samples were adjusted to the same concentration of chromatin. Samples were diluted 10 times in dilution buffer (0.01% SDS, 1.1% Triton-X-100, 1.2 mM EDTA, 16.7 mM Tris pH 8.1, 167 mM NaCl) followed by a 2-h preclearing with 500 μl of previously blocked protein-A and protein-G beads (Sigma). Blocking was achieved by incubating the beads with 0.5 mg ml^−1^ of Ultrapure BSA and 0.2 mg ml^−1^ of salmon sperm DNA (Invitrogen) for 3 h at 4 °C. Chromatin (100 μl) was kept for inputs. Precleared samples (20 μg of chromatin per ChIP) were incubated overnight at 4 °C with 1 μg of antibody. A Mock sample without antibody was processed in parallel to assess the ChIP efficiency. Immune complexes were then recovered by incubating the samples with 20 μl of blocked protein-A/protein-G beads for 2 h at 4 °C on a rotating wheel. Beads were washed once in dialysis buffer (2 mM EDTA, 50 mM Tris pH 8, 0.2% Sarkosyl), four times in wash buffer (100 mM Tris pH 8.8, 500 mM LiCl, 1% NP40, 1% NaDoc) and twice in TE buffer (10 mM Tris pH 8, 1 mM EDTA). The bead/chromatin complexes were resuspended in 200 μl of TE buffer and incubated 30 min at 37 °C with 10 μg of RNase A (Abcam), as well as input DNA. Formaldehyde crosslink was reversed in the presence of 0.2% SDS at 70 °C overnight. After a 2-h proteinase K (0.2 mg ml^−1^) treatment at 45 °C, immunoprecipitated and input DNA were purified with phenol/chloroform, precipitated and analysed by qPCR.

### Immunoprecipitations followed by RNA incubation

H2A.Z or p400-containing complexes were purified by immunoprecipitations from 100 μl of HeLa nuclear extracts (purchased from Computer Cell Culture Center, prepared according to the classical Dignam protocol) diluted 10 times in IP buffer (20 mM Tris pH 8.0, 150 mM NaCl, 5 mM MgCl_2_, 0.1% NP40) supplemented with 20 U of DNase I (Epicentre) using 6 μg of antibodies and incubated at 4 °C over night. Protein-A/G (Sigma) beads were then added for 2 h. After five washes with IP buffer, beads were resuspended in 50 μl of IP buffer and 10 μg of yeast tRNA (Ambion) and incubated 30 min at 4 °C with shaking. After two washes with IP buffer, the saturated beads were resuspended in 50 μl of IP buffer supplemented with 80 U of RNase Inhibitor (Epicentre). Total RNA (4 μg) purified from senescent WI38 cells was added and incubated 2 h at 4 °C under shaking. After four washes with IP buffer, RNA bound to beads were purified using the MasterPure RNA Purification Kit (Epicentre Biotechnologies) supplemented with Baseline-ZERO DNase (Epicentre), according to the manufacturer’s recommendations. The purified RNAs as well as input RNA (10%) were analysed by qRT–PCR.

### Cell fractionation experiments

Cell pellets (10^6^ cells) were resuspended in 50 μl of lysis buffer (10 mM Tris pH 8.0, 10 mM NaCl, 2 mM MgCl_2_ supplemented with protease inhibitor cocktail (Roche)) and incubated at 4 °C for 5 min. NP40 was then added to a final concentration of 0.5% and incubation was continued for 10 min at 4 °C. Samples were centrifuged at 1,000*g* for 5 min. The supernatant was collected and represented the cytoplasmic fraction. The pellet was resuspended in 10 μl of buffer 3 (20 mM Hepes pH 7.9, 420 mM NaCl, 1.5 mM MgCl2, 0.2 mM EDTA, 10% Glycerol) supplemented with 1 μl of RNase inhibitor (Epicentre) and with protease inhibitor cocktail (Roche) and incubated 30 min at 4 °C. Samples were centrifuged at full speed. The supernatant and the pellet were collected and represented soluble nuclear fraction and chromatin fraction, respectively. The three fractions were then subjected to total RNA extraction using the MasterPure RNA Purification Kit (Epicentre Biotechnologies) supplemented with Baseline-ZERO DNase (Epicentre), according to the manufacturer’s recommendations. RNA concentrations were then measured and 200 ng of RNA was then analysed by qRT–PCR as described above.

### Antibodies

Antibodies against p15 (C20, Santa-Cruz), p16 (EPR1473, Epitomics), p21 (C19, Santa-Cruz), GAPDH (MAB 374, Chemicon International), PanAcH4 (06-866, Millipore), H3K4me3 (pAb-003, Diagenode), HMGA1 (ab4078, Abcam), H2A.Z (ab4174, Abcam), p400 (ab5201, Abcam), RNA PolII S2P (ab5095, Abcam), SYDE2 (HPA027138, Sigma), DDAH1 (ab82908, Abcam) and CYR61 (sc-374129, Santa-Cruz) were purchased.

### siRNA and primers

siRNAs were designed to favour guide strand upload into the RNA-induced silencing complex^45^ and purchased from Eurogentec (VAD1, VAD2 and control siRNAs) or from Dharmacon (ON-TARGETplus VAD1 or VAD2). Others siRNAs were purchased from Dharmacon: p21/CDKN1A (siGENOME SMARTpool-Human CDKN1A, M-003471-00), p16 (ref. 21), DDAH1 (ON-TARGETplus SMARTpool, LU-008528-01), CYR61 (ON-TARGETplus SMARTpool, L-004263-00), SYDE2 (ON-TARGETplus SMARTpool, L-021617-01) and controls (siGENOME Non-Targeting siRNA pool #1, D-001206-13 and ON-TARGETplus Non-targeting siRNA #1 or #4, D-001810-01).

Sequences of siRNA, primers used in strand-specific RT experiments and primers used for qPCR are described in Supplementary Tables 1–3, respectively.

## Author contributions

S.L. designed, performed and analysed the experiments. E.N. designed, performed and analysed the experiments, supervised the project and wrote the paper. D.T. supervised the project and wrote the paper. S.B., M.A. and J.-Y.T. developed analytical tools. C.M. generated the WI38-derived cell lines and siRNA knockdown protocols. G.S.L. and P.K. extensively shared and discussed the data. C.V. and C.R. did the RNA- and DNA-FISH experiments.

## Additional information

**How to cite this article:** Lazorthes, S. *et al*. A vlincRNA participates in senescence maintenance by relieving H2AZ-mediated repression at the *INK4* locus. *Nat. Commun.* 6:5971 doi: 10.1038/ncomms6971 (2015).

**Accession codes:** Microarray data have been deposited at the Gene Expression Omnibus under the accession number GSE53578.

## Supplementary Material

Supplementary Figures, Supplementary Tables and Supplementary References Supplementary Figures 1-16 and Supplementary Tables 1-3 and Supplementary References

Supplementary Data 1 List of all transcript domains whose expression changed during oncogene-induced senescence

Supplementary Data 2 List of all antisense transcript domains whose expression changed during oncogene-induced senescence

Supplementary Data 3 List of the putative vlinc transcript domains whose expression changed during oncogene-induced senescence

## Figures and Tables

**Table 1:** Analysis of strand-specific transcriptome changes during senescence on human chromosomes 1 and 6.

**Transcript domains**	**Total**	**Intergenic**	**Gene(s)**	**Sense**	**Antisense**	**Vlincs**
Repressed	762	57	705	622	219	30
Activated	379	35	344	289	134	41
Total	1141	92	1049	911	353	71

Total number of differentially expressed transfrags (transcript domains) whose expression is either repressed or activated during senescence as well as the number of differentially expressed transfrags in different categories: totally intergenic, overlapping (partially or totally) with at least one annotated gene from RefSeq database (Genes), partially or totally sense or antisense to at least one annotated gene, or containing at least 50 kb of sequences that do not correspond to an annotated RNA (‘Vlincs’). Note that the total number of differentially expressed transfrags overlapping genes is lower than the sum of the number of differentially expressed transfrags sense or antisense to genes, because some differentially expressed transfrags are sense and/or antisense to more than one gene.

**Table 2:** Number and proportion (parentheses) of genes overlapping partially or totally with at least one differentially expressed transfrag (sense or antisense) whose expression changes during senescence.

**Genes**	**Sense**	**Antisense**
Repressed	646 (18.1%)	239 (6.7%)
Activated	311 (8.7%)	160 (4.5%)
Total	957 (26.8%)	399 (11.2%)

Gene number of chromosomes 1 and 6=3,570.

## References

[b1] Bartkova J. . Oncogene-induced senescence is part of the tumorigenesis barrier imposed by DNA damage checkpoints. Nature 444, 633–637 (2006). 17136093 10.1038/nature05268

[b2] Di Micco R. . Oncogene-induced senescence is a DNA damage response triggered by DNA hyper-replication. Nature 444, 638–642 (2006). 17136094 10.1038/nature05327

[b3] Xue W. . Senescence and tumour clearance is triggered by p53 restoration in murine liver carcinomas. Nature 445, 656–660 (2007). 17251933 10.1038/nature05529 PMC4601097

[b4] Campisi J. & d'Adda di Fagagna F. Cellular senescence: when bad things happen to good cells. Nat. Rev. Mol. Cell Biol. 8, 729–740 (2007). 17667954 10.1038/nrm2233

[b5] Narita M. . Rb-mediated heterochromatin formation and silencing of E2F target genes during cellular senescence. Cell 113, 703–716 (2003). 12809602 10.1016/s0092-8674(03)00401-x

[b6] Narita M. . A novel role for high-mobility group a proteins in cellular senescence and heterochromatin formation. Cell 126, 503–514 (2006). 16901784 10.1016/j.cell.2006.05.052

[b7] Chandra T. . Independence of repressive histone marks and chromatin compaction during senescent heterochromatic layer formation. Mol. Cell 47, 203–214 (2012). 22795131 10.1016/j.molcel.2012.06.010 PMC3701408

[b8] Fridman A. L. & Tainsky M. A. Critical pathways in cellular senescence and immortalization revealed by gene expression profiling. Oncogene 27, 5975–5987 (2008). 18711403 10.1038/onc.2008.213 PMC3843241

[b9] Benhamed M., Herbig U., Ye T., Dejean A. & Bischof O. Senescence is an endogenous trigger for microRNA-directed transcriptional gene silencing in human cells. Nat. Cell Biol. 14, 266–275 (2012). 22366686 10.1038/ncb2443 PMC5423543

[b10] Abdelmohsen K. . Senescence-associated lncRNAs: senescence-associated long noncoding RNAs. Aging Cell 12, 890–900 (2013). 23758631 10.1111/acel.12115 PMC3773026

[b11] Magistri M., Faghihi M. A., St Laurent G. 3rd & Wahlestedt C. Regulation of chromatin structure by long noncoding RNAs: focus on natural antisense transcripts. Trends Genet. 28, 389–396 (2012). 22541732 10.1016/j.tig.2012.03.013 PMC3768148

[b12] Djebali S. . Landscape of transcription in human cells. Nature 489, 101–108 (2012). 22955620 10.1038/nature11233 PMC3684276

[b13] Bernstein B. E. . An integrated encyclopedia of DNA elements in the human genome. Nature 489, 57–74 (2012). 22955616 10.1038/nature11247 PMC3439153

[b14] Kapranov P. . The majority of total nuclear-encoded non-ribosomal RNA in a human cell is 'dark matter' un-annotated RNA. BMC Biol. 8, 149 (2010). 21176148 10.1186/1741-7007-8-149 PMC3022773

[b15] St Laurent G. 3rd . VlincRNAs controlled by retroviral elements are a hallmark of pluripotency and cancer. Genome Biol. 14, R73 (2013). 23876380 10.1186/gb-2013-14-7-r73 PMC4053963

[b16] Vallot C. . XACT, a long noncoding transcript coating the active X chromosome in human pluripotent cells. Nat. Genet. 45, 239–241 (2013). 23334669 10.1038/ng.2530

[b17] Faghihi M. A. & Wahlestedt C. Regulatory roles of natural antisense transcripts. Nat. Rev. Mol. Cell Biol. 10, 637–643 (2009). 19638999 10.1038/nrm2738 PMC2850559

[b18] Hall I. M. . Establishment and maintenance of a heterochromatin domain. Science 297, 2232–2237 (2002). 12215653 10.1126/science.1076466

[b19] Maison C. . SUMOylation promotes de novo targeting of HP1alpha to pericentric heterochromatin. Nat. Genet. 43, 220–227 (2011). 21317888 10.1038/ng.765

[b20] Volpe T. A. . Regulation of heterochromatic silencing and histone H3 lysine-9 methylation by RNAi. Science 297, 1833–1837 (2002). 12193640 10.1126/science.1074973

[b21] Jeanblanc M. . Parallel pathways in RAF-induced senescence and conditions for its reversion. Oncogene 31, 3072–3085 (2012). 22020327 10.1038/onc.2011.481

[b22] St Laurent G. 3rd . Intronic RNAs constitute the major fraction of the non-coding RNA in mammalian cells. BMC Genomics 13, 504 (2012). 23006825 10.1186/1471-2164-13-504 PMC3507791

[b23] Wu J. Q. . Systematic analysis of transcribed loci in ENCODE regions using RACE sequencing reveals extensive transcription in the human genome. Genome Biol. 9, R3 (2008). 18173853 10.1186/gb-2008-9-1-r3 PMC2395237

[b24] Mahmoudi S. . Wrap53, a natural p53 antisense transcript required for p53 induction upon DNA damage. Mol. Cell 33, 462–471 (2009). 19250907 10.1016/j.molcel.2009.01.028

[b25] Hayashi T. . Nitric oxide and endothelial cellular senescence. Pharmacol. Ther. 120, 333–339 (2008). 18930078 10.1016/j.pharmthera.2008.09.002

[b26] Leiper J. . Disruption of methylarginine metabolism impairs vascular homeostasis. Nat. Med. 13, 198–203 (2007). 17273169 10.1038/nm1543

[b27] Zeisel A. . Coupled pre-mRNA and mRNA dynamics unveil operational strategies underlying transcriptional responses to stimuli. Mol. Syst. Biol. 7, 529 (2011). 21915116 10.1038/msb.2011.62 PMC3202801

[b28] Ingolia N. T., Lareau L. F. & Weissman J. S. Ribosome profiling of mouse embryonic stem cells reveals the complexity and dynamics of mammalian proteomes. Cell 147, 789–802 (2011). 22056041 10.1016/j.cell.2011.10.002 PMC3225288

[b29] Malumbres M. . Cellular response to oncogenic ras involves induction of the Cdk4 and Cdk6 inhibitor p15(INK4b). Mol. Cell. Biol. 20, 2915–2925 (2000). 10733595 10.1128/mcb.20.8.2915-2925.2000 PMC85529

[b30] Collado M. . Tumour biology: senescence in premalignant tumours. Nature 436, 642 (2005). 16079833 10.1038/436642a

[b31] Contrepois K., Thuret J. Y., Courbeyrette R., Fenaille F. & Mann C. Deacetylation of H4-K16Ac and heterochromatin assembly in senescence. Epigenet. Chromatin 5, 15 (2012). 10.1186/1756-8935-5-15 PMC3487866 22932127

[b32] Koerner M. V., Pauler F. M., Huang R. & Barlow D. P. The function of non-coding RNAs in genomic imprinting. Development 136, 1771–1783 (2009). 19429783 10.1242/dev.030403 PMC2847617

[b33] Latos P. A. & Barlow D. P. Regulation of imprinted expression by macro non-coding RNAs. RNA Biol. 6, 100–106 (2009). 19229135 10.4161/rna.6.2.7854 PMC2847177

[b34] Sleutels F., Zwart R. & Barlow D. P. The non-coding Air RNA is required for silencing autosomal imprinted genes. Nature 415, 810–813 (2002). 11845212 10.1038/415810a

[b35] Bracken A. P. . The Polycomb group proteins bind throughout the INK4A-ARF locus and are disassociated in senescent cells. Genes Dev. 21, 525–530 (2007). 17344414 10.1101/gad.415507 PMC1820894

[b36] Creyghton M. P. . H2AZ is enriched at polycomb complex target genes in ES cells and is necessary for lineage commitment. Cell 135, 649–661 (2008). 18992931 10.1016/j.cell.2008.09.056 PMC2853257

[b37] Tsai M. C. . Long noncoding RNA as modular scaffold of histone modification complexes. Science 329, 689–693. 20616235 10.1126/science.1192002 PMC2967777

[b38] Gevry N., Chan H. M., Laflamme L., Livingston D. M. & Gaudreau L. p21 transcription is regulated by differential localization of histone H2A.Z. Genes Dev. 21, 1869–1881 (2007). 17671089 10.1101/gad.1545707 PMC1935026

[b39] Chan H. M., Narita M., Lowe S. W. & Livingston D. M. The p400 E1A-associated protein is a novel component of the p53 —> p21 senescence pathway. Genes Dev. 19, 196–201 (2005). 15655109 10.1101/gad.1280205 PMC545875

[b40] Young A. P. . VHL loss actuates a HIF-independent senescence programme mediated by Rb and p400. Nat. Cell Biol. 10, 361–369 (2008). 18297059 10.1038/ncb1699

[b41] Beausejour C. M. . Reversal of human cellular senescence: roles of the p53 and p16 pathways. EMBO J. 22, 4212–4222 (2003). 12912919 10.1093/emboj/cdg417 PMC175806

[b42] Lapasset L. . Rejuvenating senescent and centenarian human cells by reprogramming through the pluripotent state. Genes Dev. 25, 2248–2253 (2011). 22056670 10.1101/gad.173922.111 PMC3219229

[b43] Zhang R. . Formation of MacroH2A-containing senescence-associated heterochromatin foci and senescence driven by ASF1a and HIRA. Dev. Cell 8, 19–30 (2005). 15621527 10.1016/j.devcel.2004.10.019

[b44] Fusco A. & Fedele M. Roles of HMGA proteins in cancer. Nat. Rev. Cancer 7, 899–910 (2007). 18004397 10.1038/nrc2271

[b45] Schwarz D. S. . Asymmetry in the assembly of the RNAi enzyme complex. Cell 115, 199–208 (2003). 14567917 10.1016/s0092-8674(03)00759-1

