# Supplementary material for: A vlincRNA participates in senescence maintenance by relieving H2AZ-mediated repression at the INK4 locus
Source: Nat Commun. 2015 Jan 20;6:5971. doi: 10.1038/ncomms6971 (PMC4309439; doi:10.1038/ncomms6971)
Supplement: Supplementary Figures, Supplementary Tables and Supplementary References — Supplementary Figures 1-16 and Supplementary Tables 1-3 and Supplementary References [file ncomms6971-s1.pdf]

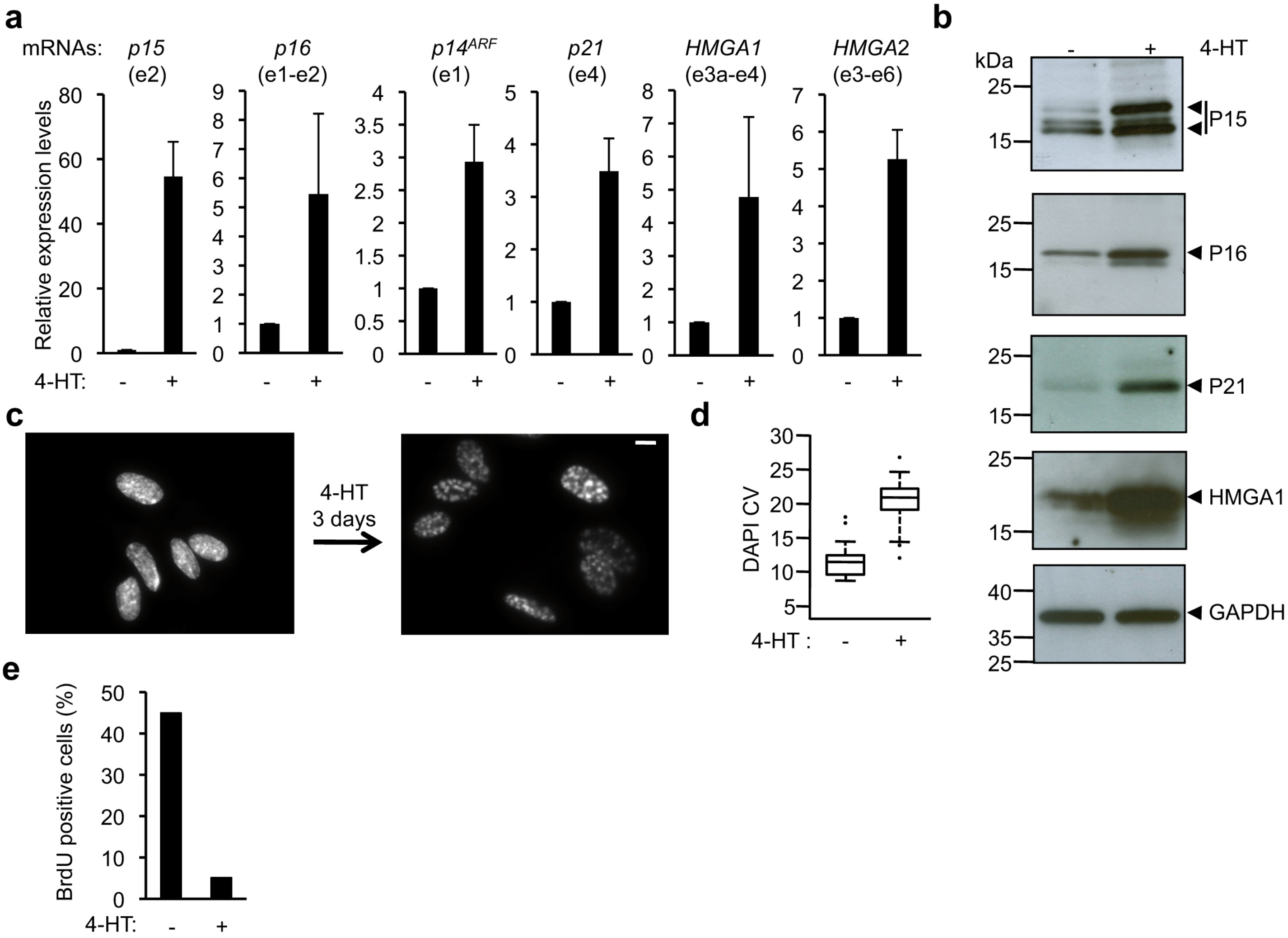

Supplementary Figure 1: Characterization of senescence induction. WI38 hTERT RAF1-ER cells were treated or not with 4-HT. 72 hours later, cells were harvested and analyzed (**a**) by qRT-PCR for the indicated mRNA expression (mean and SD from three independent experiments, relative to *GAPDH* and normalized to 1 in proliferative cells) (**b**) by Western blot for the indicated protein expression, (**c**) by DAPI staining (scale bar = 7 μm) to analyze the presence of SAHF followed by the quantification of the chromatin compaction (DAPI CV (Coefficent of variation)) (**d**) and by BrdU staining to analyze the cell proliferation arrest (**e**).

Lazorthes et al.,
Supplementary Figure 1

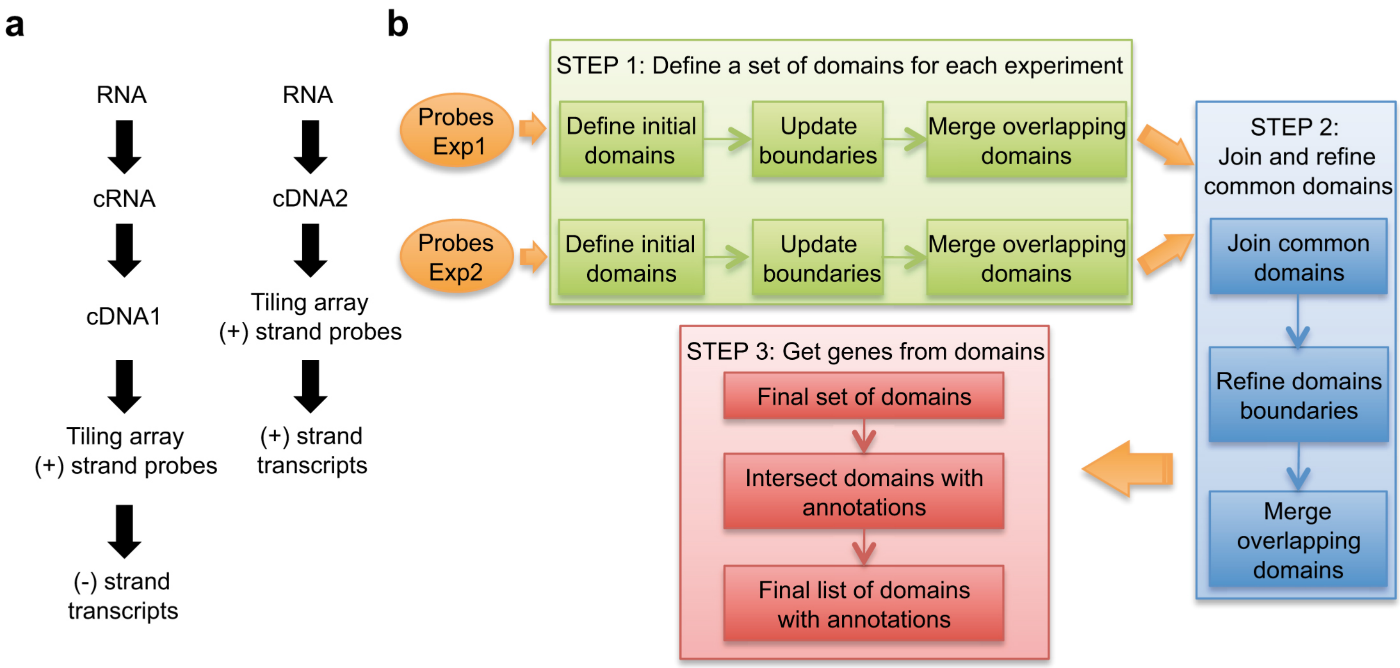

Supplementary Figure 2: Identification of RNAs from chromosomes 1 and 6 whose expression changes during senescence. (**a**) Methodology for probe preparation to obtain information on RNAs transcribed from the minus (-) and the plus (+) strand. In order to analyze the RNAs originated from plus and minus strands of DNA, two different cDNAs were synthesized: one is complementary to the RNAs (cDNA2) while the other one has the same orientation (cDNA1) as the RNAs. No amplification by PCR was performed. Each cDNA was labeled and hybridized on a tiling array (GeneChip Human Tiling 2.OR A arrays (Affymetrix), which contains probes corresponding to the (+) strand of DNA). Those two cDNAs were synthesized from proliferative cells and senescent cells. Plus strand RNA changes were monitored by the ratio cDNA2 in senescence / cDNA2 in proliferation, and minus strand RNA changes were monitored by the ratio cDNA1 in senescence / cDNA1 in proliferation. This protocol was derived from a strand specific expression protocol on exon arrays[1] and already used in Iacovoni et al.[2]. (**b**) Schematic representation of the algorithm used for the analysis of genome wide data. Note that data from two independent experiments were used in the analysis. All domains identified at the beginning of step 3 (« Final set of domains ») are listed in Supplementary Data 1. They represent RNAs whose expression changes during senescence (p-value < 0.025 as calculated through data randomization).

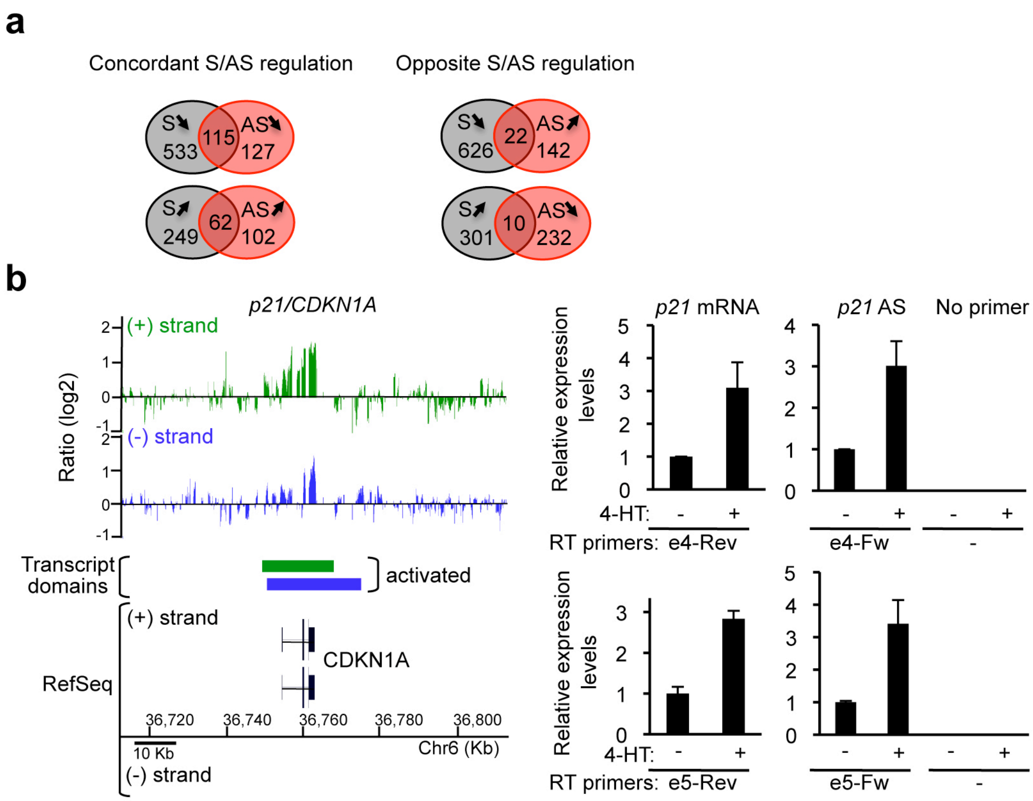

Supplementary Figure 3: Analysis of the extent of co-regulation between antisense and sense transcription in senescence. (**a**) Venn diagrams representing the extent of co-regulation between sense (S) and antisense (AS) transcription (from the genes presented in Table 2) during senescence when both change in the same or opposite way. Note that the total number of genes overlapping differentially expressed transfrags in Table 2 is lower than the sum of the number of genes overlapping differentially expressed transfrags that change in a similar or opposite way, because some genes overlap more than one differentially expressed transfrag. (**b**) Example of the *p21/CDKN1A* gene presenting a concordant regulation of its sense and antisense transcription. Left panel: schematic representation of the *p21/CDKN1A* gene including tiling array data (log2 of senescence/proliferative expression ratio, each bar corresponds to the Ratio for one probe). Transcript domains (differentially expressed transfrags) identified by the algorithm are indicated. The ratios and the domains are shown in green or in blue, for the (+) strand or for the (-) strand, respectively. For each transcript variant (from RefSeq, visualized in IGB, Affymetrix), vertical and horizontal lines represent exons and introns, respectively. Tall and small vertical lines represent coding and 5' or 3' UTR sequences, respectively. Right panels: validation of sense and antisense expression changes in senescence at the *p21/CDKN1A* locus by strand-specific qRT-PCR, using primers located in exon 4 (upper panels) and exon 5 (bottom panels). Total RNAs were extracted from proliferative (-4-HT) or senescent (+4-HT) WI38 hTERT RAF1-ER cells. The mean and SD from three experiments (upper panels) or from the qPCR sample triplicates for one representative experiment out of two (bottom panels) are shown, relative to *GAPDH* and normalized to 1 in proliferative cells. Parallel experiments without primers in the RT step were performed to control for genomic DNA contamination.

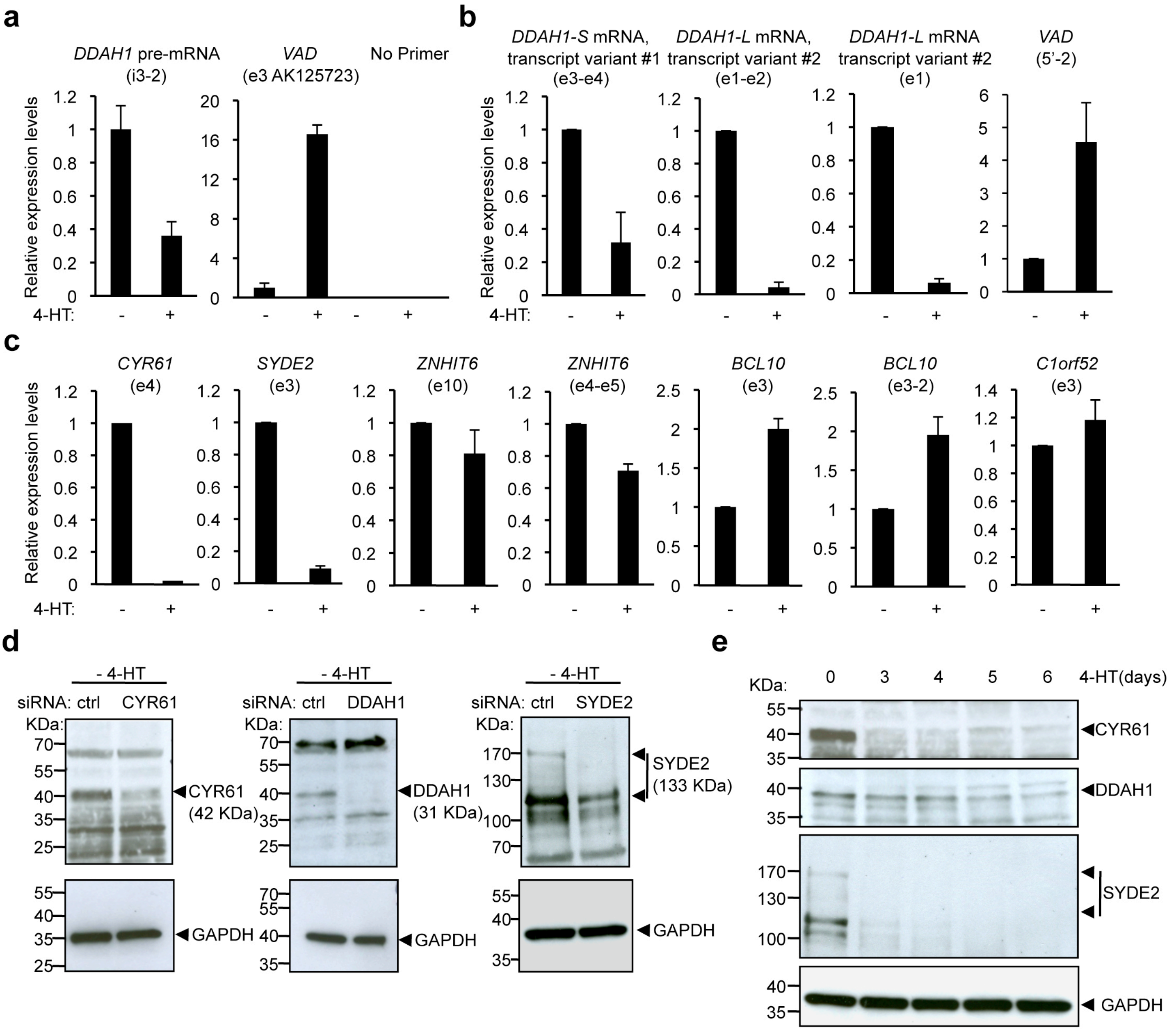

Supplementary Figure 4: Validation of senescence-linked changes of RNA and protein expression within the *VAD* locus. (**a**) WI38 hTERT RAF1-ER cells were treated or not with 4-HT for 72 hours. Total RNAs were extracted and analyzed for *DDAH1* pre-mRNA or *VAD* expression by strand-specific qRT-PCR using the indicated primers. The mean and SD from the qPCR sample triplicates for one representative experiment out of two are shown, relative to *GAPDH* and normalized to 1 in proliferative cells (- 4-HT). (**b-c**) Same as in (a), except that total RNAs were subjected to a RT step with random primers and tested by qPCR for the expression of the indicated RNA. The mean and SD from three independent experiments are shown, relative to *GAPDH* and normalized to 1 in proliferative cells. Note that the expression changes of *BCL10* and *ZNIHT6* were assessed using 2 different sets of primers because the expression changes observed by qRT-PCR were different from those observed by the tiling array analysis with only a slight decrease for *ZNIHT6* and an increase for *BCL10*. (**d**) Proliferative cells were subjected to the indicated siRNA treatment. Whole cell protein extracts were subjected to immunoblotting using the indicated antibodies. (**e**) Kinetics of the repression of DDAH1, CYR61 and SYDE2 proteins during senescence. WI38 hTERT RAF1-ER cells were induced, or not, in senescence for the indicated number of days. Whole cell protein extracts were subjected to immunoblotting using the indicated antibodies. The antibody specificity was assessed by doing siRNA experiments shown in (d).

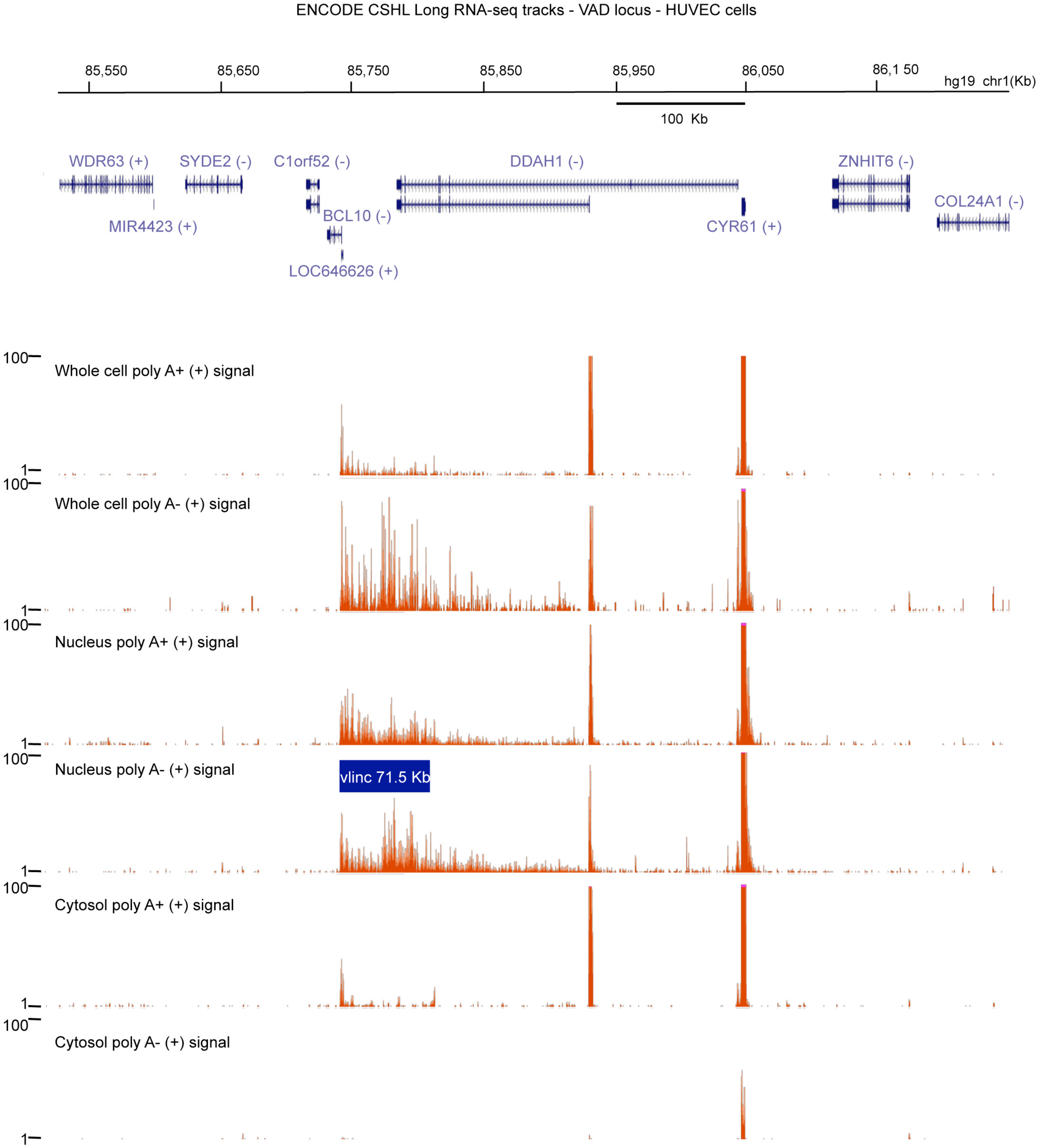

Supplementary Figure 5: *VAD* is expressed in proliferating HUVEC cells. RefSeq Genes (Human genome version 2009) and tracks ((+) strand) from HUVEC whole cell, nucleus and cytosol poly A+ or polyA- ENCODE CSHL strand-specific long RNA sequencing data are shown. Informatic analysis of HUVEC nuclear poly A- RNA data uncovered a vlincRNA partially corresponding to *VAD* (71.5 Kb, blue box). Note that this vlincRNA is detected almost exclusively in the nucleus and is enriched in polyA- RNA as compared to polyA+ RNA.

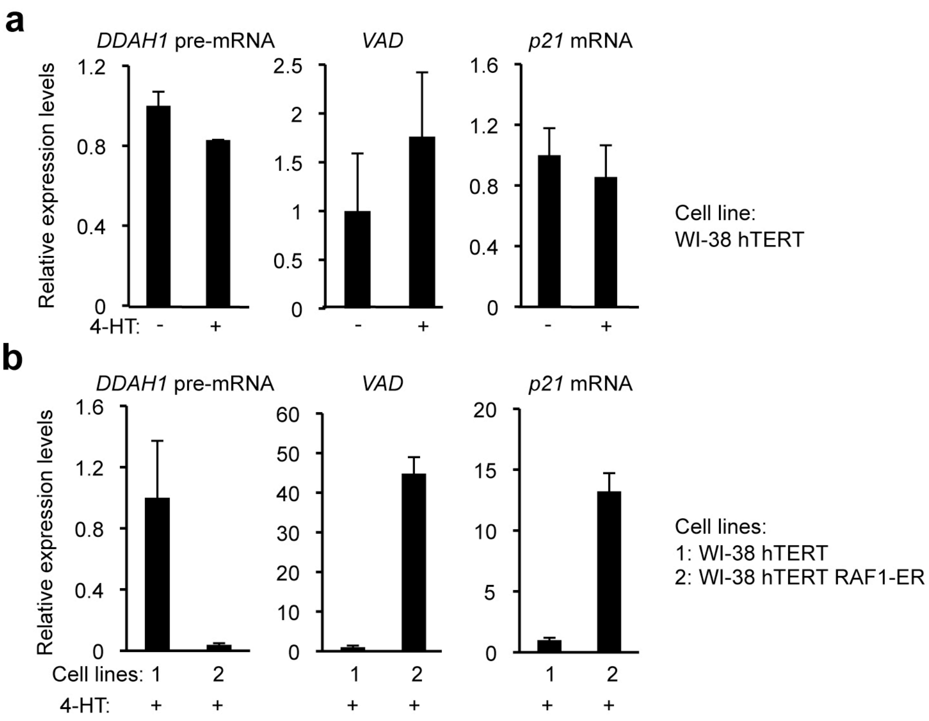

Supplementary Figure 6: Changes of RNA expression at the *VAD* locus are due to senescence and not to 4-HT treatment. (**a**) WI38 hTERT cells, which do not contain the RAF1-ER construct, were treated or not with 4-HT for 72 hours. Total RNA was extracted and subjected to strand-specific qRT-PCR analysis to monitor the expression of the indicated RNA (using i3-1 primers for *DDAH1* pre-mRNA, 3'-1 primers for *VAD* and e4 primers for *p21* mRNA). The slight changes in expression upon 4-HT addition are not reproducible and not comparable to the changes observed in the WI38 hTERT RAF1-ER cell line (Fig. 2b and Supplementary Fig. 3b). Mean and SD from the qPCR sample triplicates, relative to *GAPDH* and normalized to 1 in -4-HT sample. (**b**) Same as in (a) except that WI38 hTERT cells (cell line #1) or WI38 hTERT RAF1-ER cells (cell line #2) were both treated with 4-HT for 72 hours. Note that the differences in RNA expression between the two cell lines are comparable to the changes observed in the WI38 hTERT RAF1-ER cell line treated or not with 4-HT (Fig. 2b and Supplementary Fig. 3b). Mean and SD from the qPCR sample triplicates, relative to *GAPDH* and normalized to 1 in cell line #1.

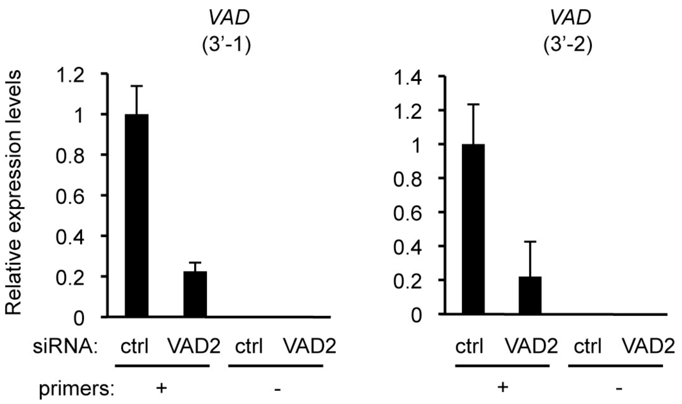

Supplementary Figure 7: *VAD* is a very long RNA of more than 200 Kb. Senescent WI38 hTERT RAF1-ER cells were transfected using the VAD2 siRNA (located at the 5' end of *VAD*) or a control siRNA (ctrl), and *VAD* expression was measured 72 hours later by strand-specific qRT-PCR using primers located at the 3' end of the *VAD* RNA (see Fig. 2a for primer location). The no primer samples show the absence of DNA contamination. The mean and SD from the qPCR sample triplicates for one representative experiment out of two are shown, relative to *GAPDH* and normalized to 1 in the siRNA control sample.

Lazorthes et al.,
Supplementary Figure 7

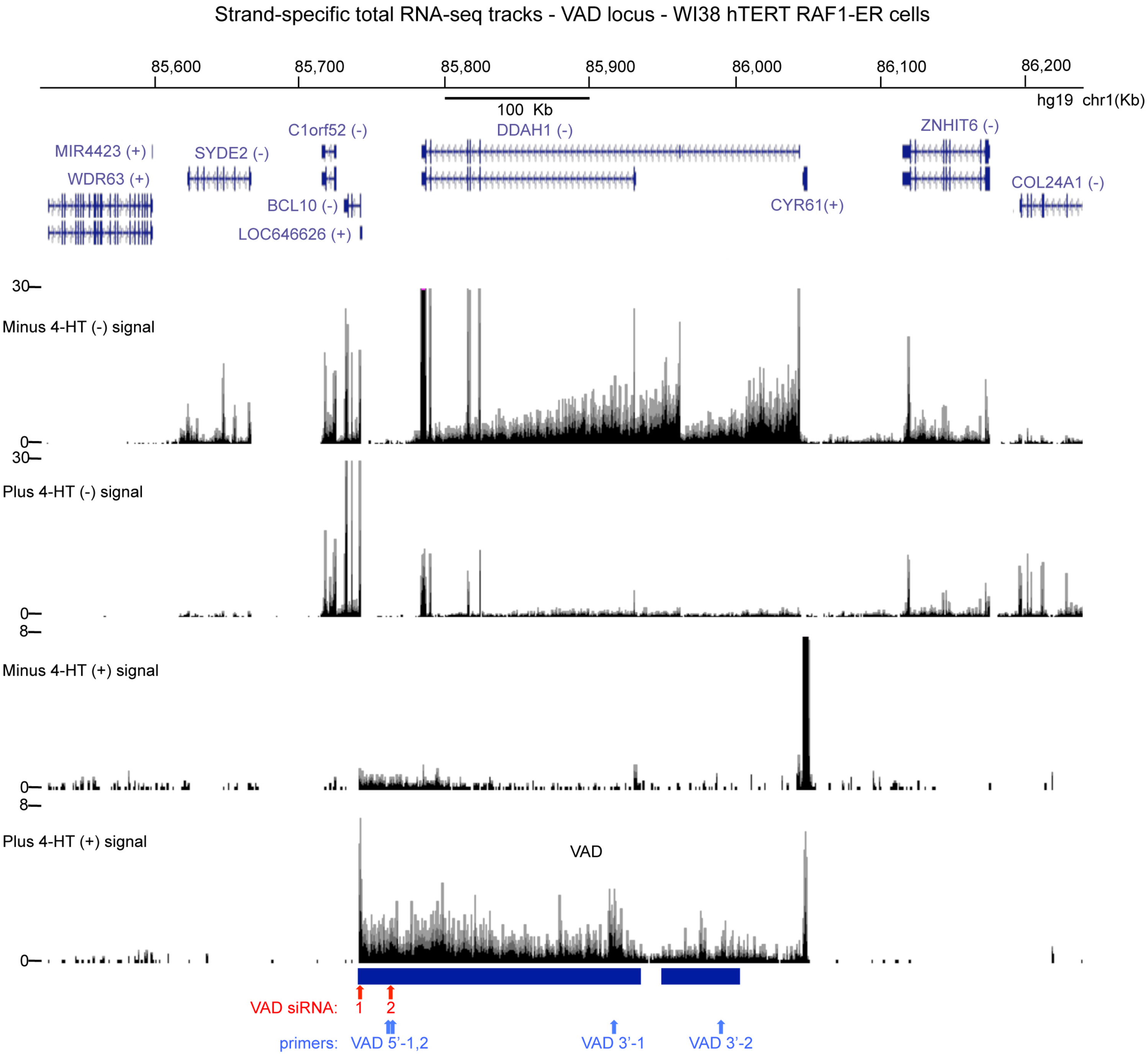

Supplementary Figure 8: Strand-specific RNA-seq of total RNAs from proliferating and senescent WI38 hTERT RAF1-ER cells. RefSeq Genes (hg19) and tracks from strand-specific total RNA-seq in WI38 hTERT RAF1-ER cells induced (plus 4-HT) or not (minus 4-HT) in senescence (Lazorthes et *al*., in preparation) visualized in UCSC are shown for both DNA strands at the *VAD* locus. VlincRNA informatic analysis uncovered two vlincRNA domains (blue boxes) corresponding to *VAD* transcript. Red and blue arrows show the locations of the siRNAs and primers, respectively, used in this study. Note in senescence the strong decrease of *DDAH1, SYDE2* and *ZNHIT6* from the (-) strand and of *CYR61* from the (+) strand, as well as the strong increase of *VAD* vlincRNA from the (+) strand. Note that there is no evidence of efficient splicing for *VAD* and of high levels of small RNAs produced from *VAD* region. First, the reads corresponding to *VAD* are roughly constant, except at the very beginning of *VAD* (in exon 1 of AK125723), and we do not observe prominent peaks elsewhere, which could correspond to high levels of small RNAs or spliced sequences such as the ones observed for classical mRNAs *e.g. DDAH1* mRNA. Second, our analysis of the efficiency of splicing on *VAD* indicates that less than 1% of the total reads correspond to spliced reads (as compared to the average splicing efficiency of all expressed genes (>200 nt), which is about 20 %).

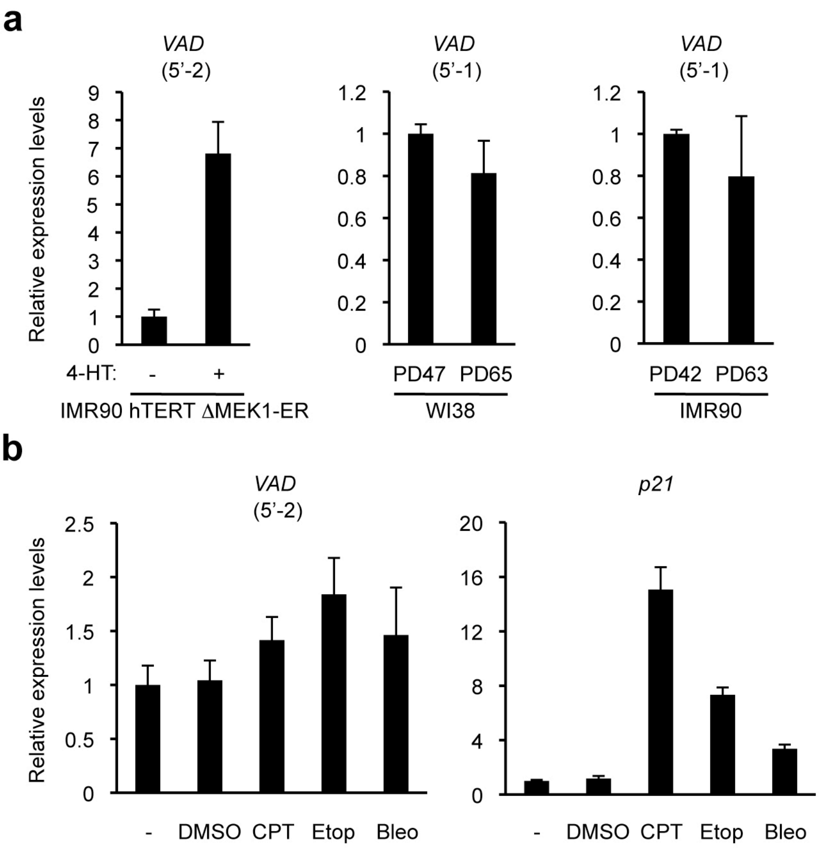

Supplementary Figure 9: *VAD* expression in MEK1-induced senescence, replicative senescence and after DNA-damaging agent treatments. (**a**) IMR90 hTERT MEK1-ER cells induced in senescence or not with 4-HT treatment, proliferating WI38 or IMR90 cells (Population doubling (PD) 47 or 42, respectively) and WI38 or IMR90 cells undergoing replicative senescence (stably arrested, PD 65 and 63, respectively) were assessed for *VAD* expression by qRT-PCR using the indicated primers. The mean and SD from the qPCR sample triplicates for one representative experiment out of two are shown, relative to *GAPDH* and normalized to 1 in proliferative cells. (**b**) *VAD* expression was analyzed from proliferating WI38 hTERT RAF1-ER cells treated or not with 1 μM camptothecin (CPT), 20 μg ml^{-1} etoposide, 1 μg ml^{-1} bleomycin or the equivalent amount of DMSO as control, for 3 days. *p21* expression was analyzed to monitor DNA-damage response induction. Mean and SD from the qPCR sample triplicates, relative to *GAPDH* and normalized to 1 in untreated cells.

Lazorthes et al.,
Supplementary Figure 9

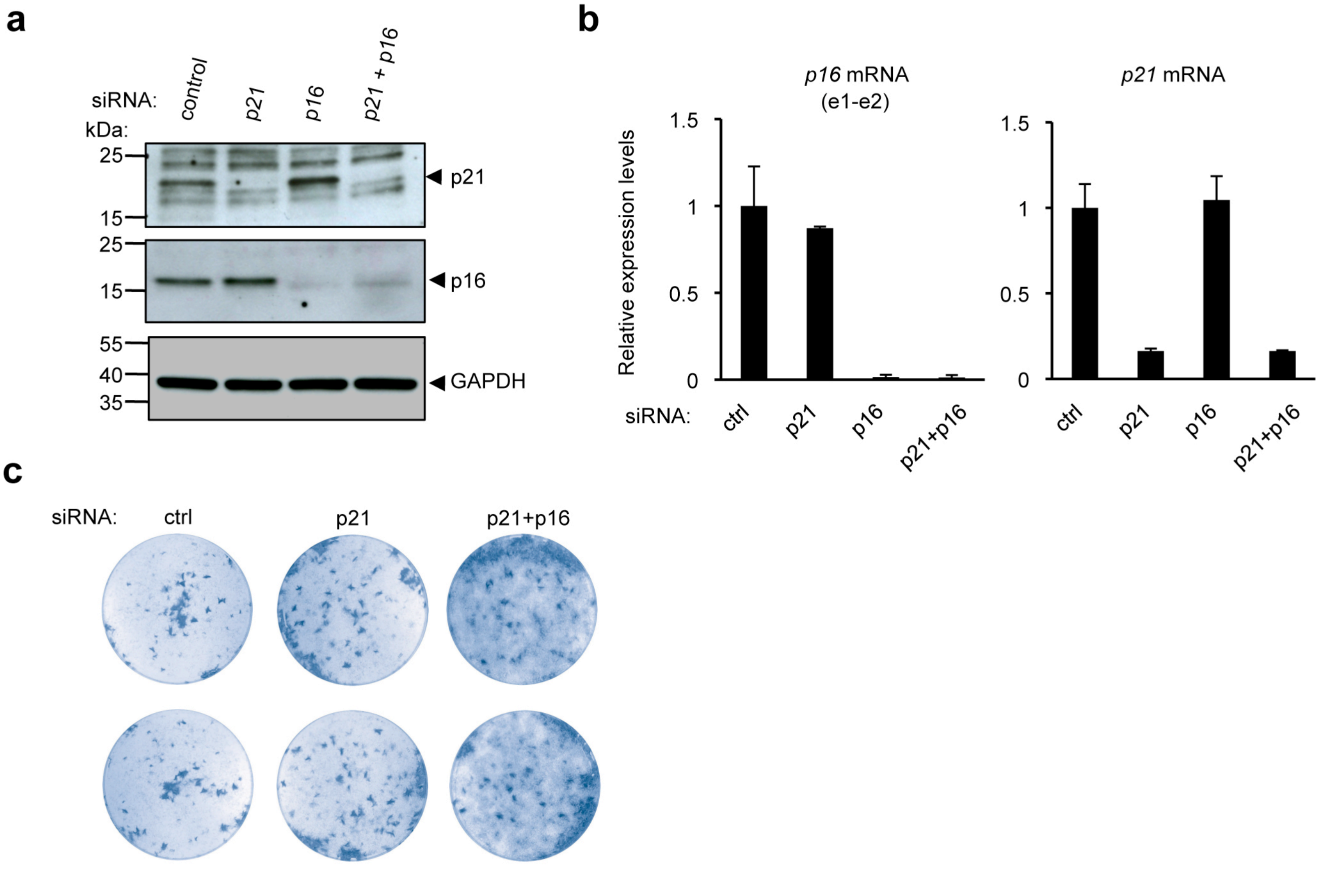

Supplementary Figure 10: Effect of p21 or both p21 and p16 depletion on senescent WI38 hTERT RAF1-ER clonogenic potential. (**a**) WI38 hTERT RAF1-ER cells were transfected using the indicated siRNAs and induced to senescence by 4-HT treatment for 72h. Total proteins were extracted and analyzed by Western blot using p21, p16 and GAPDH antibodies, as indicated. (**b**) Same as in (a) except that total RNAs were prepared and analyzed by qRT-PCR for the expression of the indicated mRNA. Mean and SD from the qPCR sample triplicates, relative to *GAPDH* and normalized to 1 in siRNA control-treated cells. (**c**) Senescent WI38 hTERT RAF1-ER cells were transfected using the indicated siRNAs, and subjected to a clonogenic assay.

Lazorthes et al.,
Supplementary Figure 10

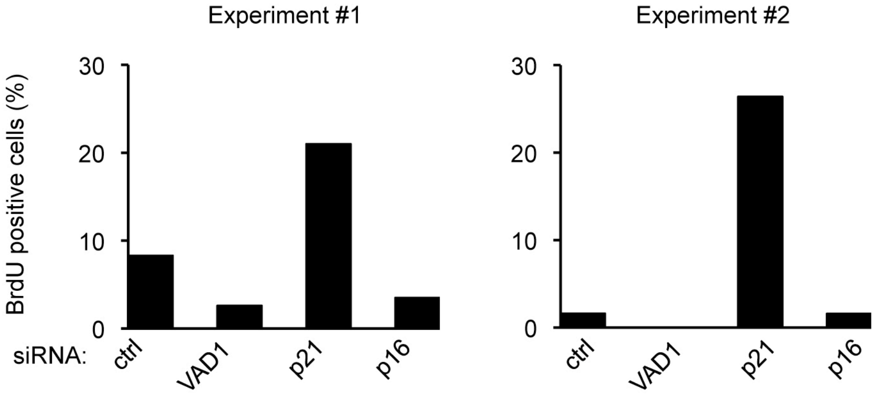

Supplementary Figure 11: ***VAD* depletion in senescent cells does not lead to an increase of BrdU-positive cells.** Senescent cells were transfected with the indicated siRNAs and subjected to BrdU staining 72 hours after transfection. BrdU-positive cells were counted, two independent experiments are shown. Note the reversion of cell proliferation arrest, measured by BrdU staining, after the depletion of *p21*, but not of *VAD* or of *p16,* whereas their depletion did reverse the compaction of SAHF (Fig. 5e) and the cell proliferation arrest measured by clonogenic assays (Fig. 3d and Supplementary Fig. 10).

Lazorthes et al.,
Supplementary Figure 11

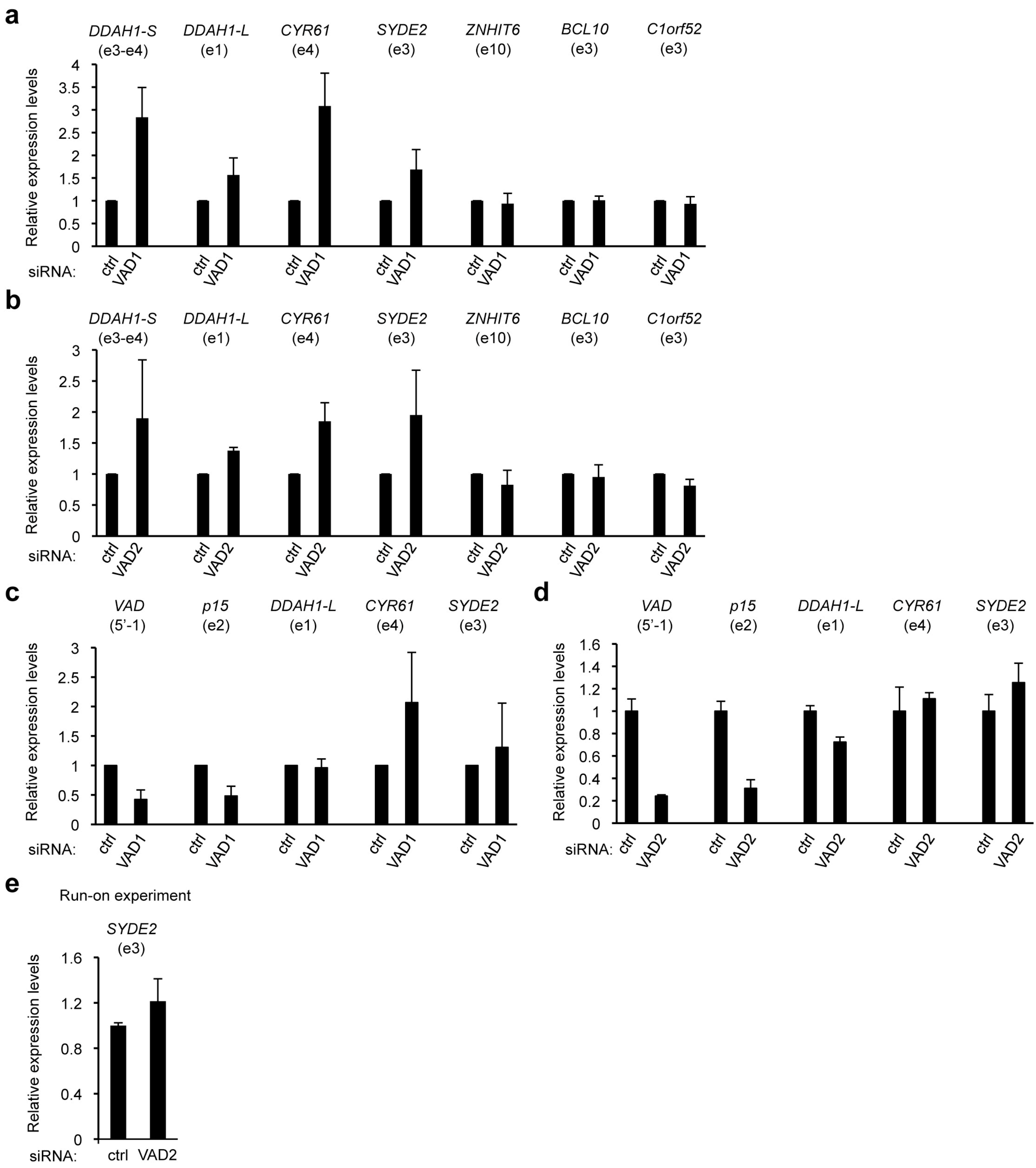

Supplementary Figure 12: **Analysis of the effect of *VAD* depletion on mRNA expression in *cis* (a-d)** Senescent WI38 hTERT RAF1-ER cells were transfected using the indicated siRNAs ((a-b) 100 nM of Control and VAD1 or VAD2 siRNAs, (c-d) 33 nM of Control and VAD1 or VAD2 siRNAs) and the expression of the indicated RNAs was analyzed by qRT-PCR 72 hours later. The mean and SD from three independent experiments (a-c) or from the qPCR sample triplicates of one representative experiment out of two (d), relative to *GAPDH* and normalized to 1 in control cells, are shown. Note that *VAD* depletion did not affect the steady-state levels of *ZNHIT6, BCL10* and *C1orf52* mRNAs and that its effect on the steady-state levels of *DDAH1, CYR61* and *SYDE2* mRNAs was not consistent from one experiment to the other, whereas *VAD* depletion efficiency and its effect on the expression of *INK4* genes were comparable (see also Fig. 5c and Supplementary Fig. 14). (**e**) Same as in (a-b) except that Run-on experiment was performed and the levels of nascent *SYDE2* RNAs were assessed after *VAD* depletion. Note that *VAD* depletion did not lead to significant increase of *SYDE2* nascent RNA levels. Mean and SD from the qPCR sample triplicates of one representative experiment out of two are shown (relative to *GAPDH* and normalized to 1 in control cells).

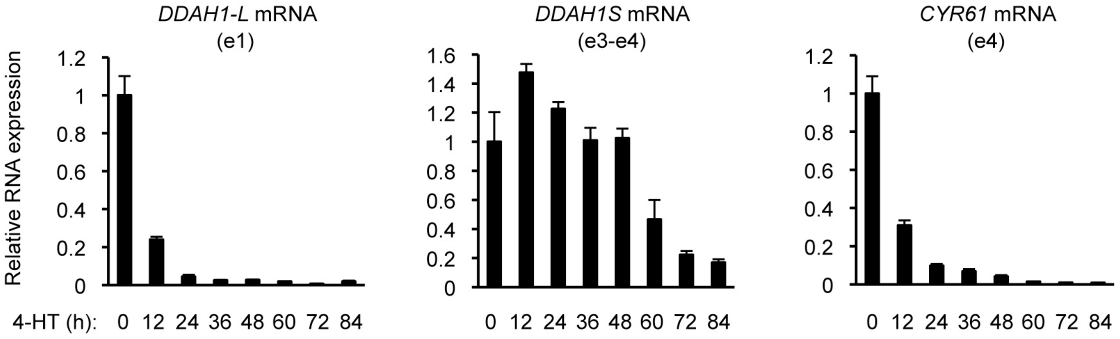

Supplementary Figure 13: Kinetics of the repression of *DDAH1* and *CYR61* mRNAs during senescence induction. WI38 hTERT RAF1-ER cells were induced to senescence, or not, for the indicated time. Total RNA was analyzed for the expression of the indicated mRNA by qRT-PCR. The mean and SD from the qPCR sample triplicates for one representative experiment out of two are shown, relative to *GAPDH* and normalized to 1 in proliferative cells (0h of 4-HT).

Lazorthes et al.,
Supplementary Figure 13

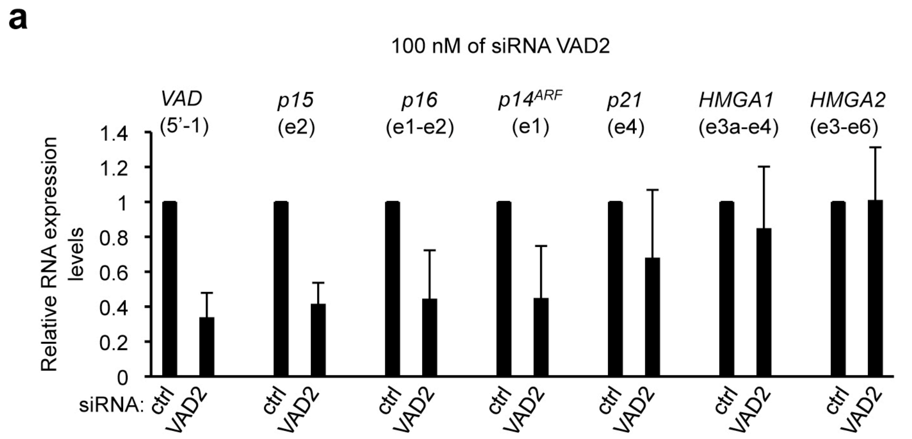

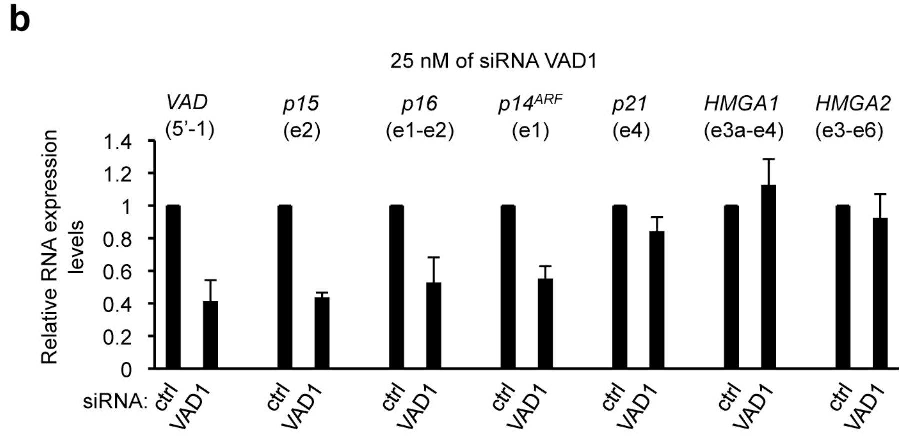

Supplementary Figure 14: Depletion of *VAD* using siRNAs primary affects the expression of *p15*, *p16* and *p14^{ARF}* *INK4* genes. Senescent WI38 hTERT RAF1-ER cells were transfected using the indicated siRNAs ((a) 100 nM of VAD2 or control siRNAs and (b) 25 nM of VAD1 or control siRNAs) and the expression of the indicated RNAs was analyzed by qRT-PCR 72 hours later. The mean and SD from three independent experiments are shown, relative to *GAPDH* and normalized to 1 in siRNA control-treated cells.

Lazorthes et al.,
Supplementary Figure 14

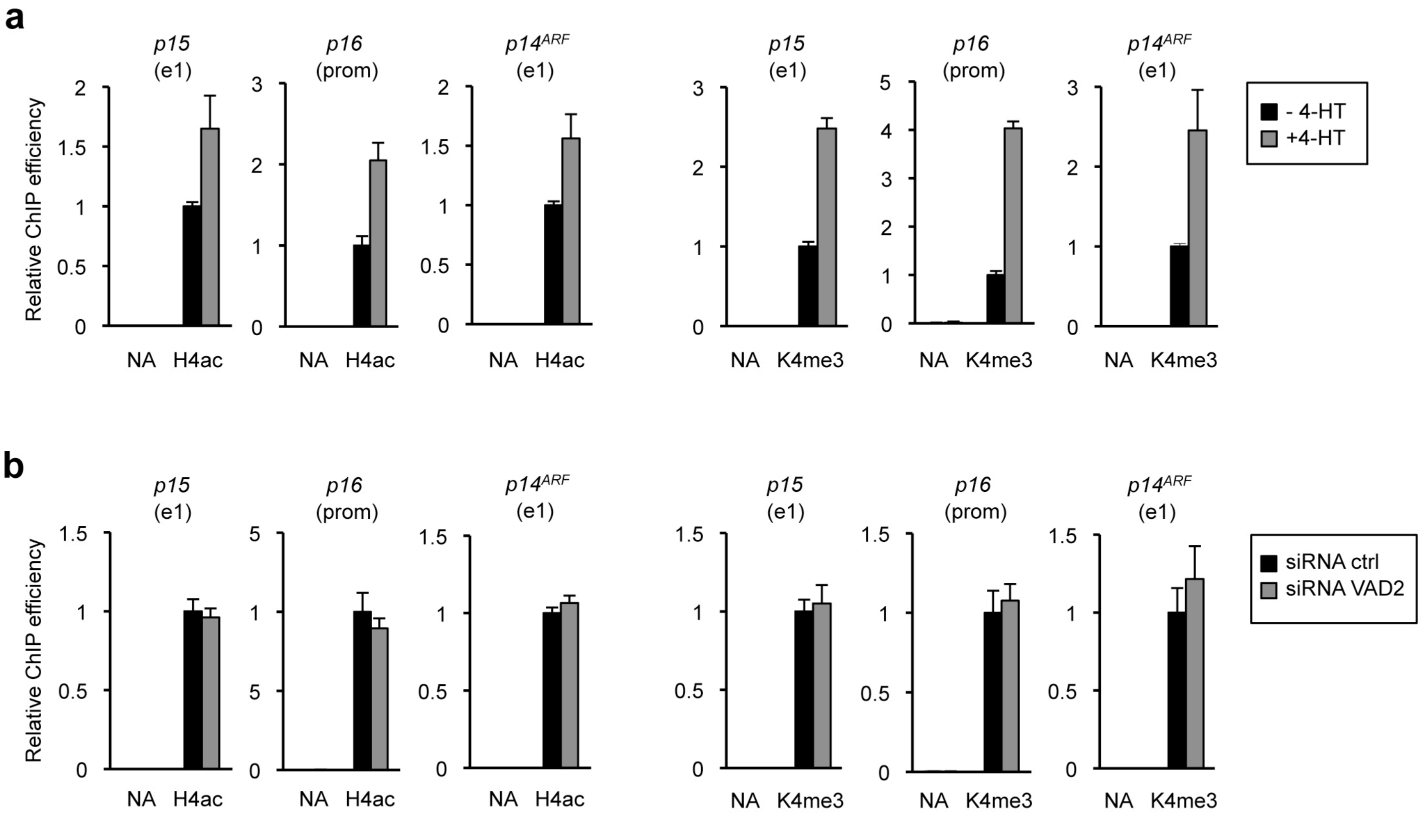

Supplementary Figure 15: *VAD* depletion does not affect H3K4me3 and H4ac at *INK4* promoters. (**a**) Chromatin prepared from WI38 hTERT RAF1-ER cells, treated or not with 4-HT for 72 hours, was immunoprecipitated with the anti H3K4me3 and H4ac antibodies or without antibody as a control (NA). The amount of the indicated sequence in the immunoprecipitates and the inputs was quantified by qPCR. For each indicated sequence, the enrichment of the ChIP H4ac or H3 K4me3 calculated relative to the input was normalized to the enrichment of H3. The ratio between the indicated sequence enrichment and a control sequence (*U6*) was then performed. A representative experiment out of two is shown (mean and SD from the qPCR sample triplicates). (**b**) Same as (a) except that chromatin was prepared from senescent WI38 hTERT RAF1-ER cells transfected with the indicated siRNAs.

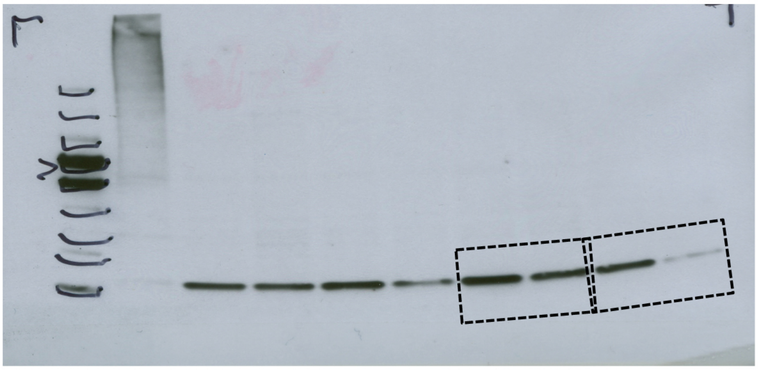

WB anti p16

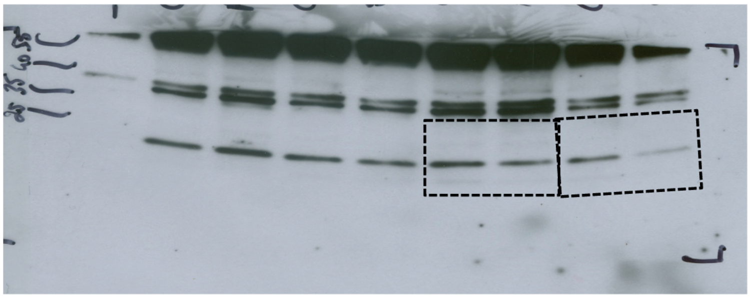

WB anti p21

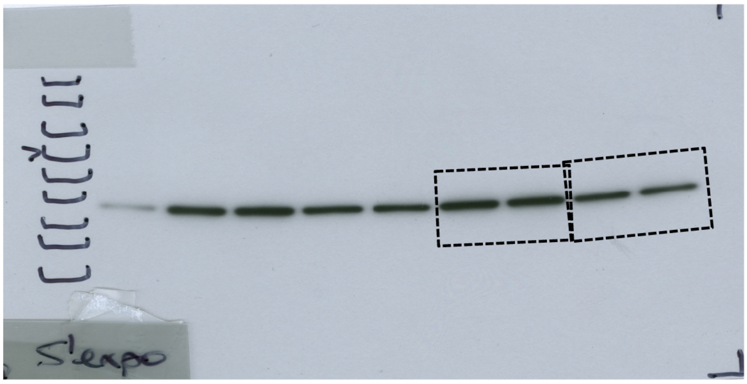

WB anti GAPDH

Supplementary Figure 16: Uncropped scans of Western blots shown in Fig. 5d

Lazorthes et al.,
Supplementary Figure 16

Supplementary Table 1: Sequences of custom siRNAs used in this study

siRNA Target	Passenger Sequence (5'-3')	Guide Sequence (5'-3')
Control #1	ACUCAAACUCACGAAGGAC-UU	P-UUCCUUCGUGAGUUUGAGU-UU
Control #2	GUCAGAGUAUCAUACGUAC-UU	P-UUACGUAUGAUACUCUGAC-UU
VAD1 (e2 AK125723)	CUACUAUGGCGCUGUGUAC-UU	P-UUACACAGCGCCAUAGUAG-UU
VAD2	GAACGAAAGUUUACAGAAC-UU	P-UUUCUGUAAACUUUCGUUC-UU
VAD1 (ON-TARGETplus Dharmacon)	CUACUAUGGCGCUGUGUAA-UU	P-UUACACAGCGCCAUAGUAG-UU
VAD2 (ON-TARGETplus Dharmacon)	GAACGAAAGUUUACAGAAA-UU	P-UUUCUGUAAACUUUCGUUC-UU
p16/CDKN2A[3,4] (ON-TARGET Dharmacon)	CCAACGCACCGAAUAGUUA-UU	P-UAACUAUUCGGUGCGUUGG-UU
H2A.Z	GUAGUGGGUUUUGAUUGAG-UU	P-CUCAAUCAAAACCCACUAC-UU

Supplementary Table 2: Sequences of primers used for strand specific RT

Strand-specific RT Target	RT	Sequence (5'-3')	qPCR primers
p21/CDKN1A e4 mRNA	RV	TGGATGCAGCCCGCCATTAG	p21/CDKN1A e4 RV and FW
p21/CDKN1A e4 AS	FW	TCAGAACCCATGCGGCAGCA	p21/CDKN1A e4 RV and FW
p21/CDKN1A e5 mRNA	RV1	GGTGAATTTCATAACCGCCTG	p21/CDKN1A e5 RV2 and FW2
p21/CDKN1A e5 AS	FW1	ATGAGAGGTTCCTAAGAGTGC	p21/CDKN1A e5 RV2 and FW2
DDAH1 pre-mRNA i1	RV1	GTGTCAGGGAATTGCTGTATGA	DDAH1 pre-mRNA i1 RV2 and FW2
DDAH1 pre-mRNA i3-1	RV	ACCTGAAAACTACATGAAGCACA	DDAH1 pre-mRNA i3-1 RV and FW
DDAH1 pre-mRNA i3-2	RV	TCCCTGCTGAAATACCTTAGTG	DDAH1 pre-mRNA i3-2 RV and FW
VAD 3'-1	RV	CAAACACACCTCTTCTCCACC	VAD 3'-1 RV and FW
VAD 3'-2	RV1	CCTAGTTCACATTGGTGAATGG	VAD 3'-2 RV2 and FW2
VAD e3 AK125723	RV	TCGGTGGCTTTGTGCATCATTT	VAD e3 AK125723 RV and FW

Supplementary Table 3: Sequences of primers used for qPCR

qPCR Target	qPCR	Sequence (5'-3')
p21/CDKN1A e4	RV	TGGATGCAGCCCGCCATTAG
	FW	TCAGAACCCATGCGGCAGCA
p21/CDKN1A e5	RV2	TGACAGCGATGGGAAGGAGC
	FW2	ATTTAAAGCCTCCTCATCCCG
DDAH1 pre-mRNA i1	RV2	ACACACTCCATACCCTTGAACA
	FW2	TTGTTACGCCATCACTCAGAAG
DDAH1 pre-mRNA i3-1	RV	ACCTGAAAACTACATGAAGCACA
	FW	CAAACACACCTCTTCTCCACC
DDAH1 pre-mRNA i3-2	RV	TCCCTGCTGAAATACCTTAGTG
	FW	TCGGTGGCTTTGTGCATCATTT
VAD 3'-1	RV	CAAACACACCTCTTCTCCACC
	FW	ACCTGAAAACTACATGAAGCACA
VAD 3'-2	RV2	TTGTTACGCCATCACTCAGAAG
	FW2	ACACACTCCATACCCTTGAACA
VAD e3 AK125723	RV	TCGGTGGCTTTGTGCATCATTT
	FW	TCCCTGCTGAAATACCTTAGTG
Intg up DUSP10	RV	GCATCCTTCTACCTATGATCTG
	FW	CAGGTAGGAACCATTATGATCC
Intg down HTR1B	RV	CACATCTAAGAGCAACCTCAG
	FW	CGACTGCTGGTTGAAGTTTTG
DDAH1-S mRNA (e3-e4)	RV	ATGCTTCTTTCATCATGTCAACC
	FW	GACTGCGTCTTCGTGGAGGA
DDAH1-L mRNA (e1-e2)	RV	GAGCTCCTTTTCCATACAGATG
	FW	ACAGCCTCTCCATATACCATG
DDAH1-L mRNA (e1)	RV	TGTCCGTCAACTTGGACTGAT
	FW	ACAGCCTCTCCATATACCATG
VAD 5'-1 (i2 AK125723)	RV	AGGGCCTGAGAAAACTCTTGG
	FW	GAAATAGTCATCGCAGGAGGC
VAD 5'-2 (i2 AK125723)	RV	GGCAGCTTCCGTATCTTTGG
	FW	GCCTTACACAGGGTCACAGT
e1 AK125728	FW	CGTTTCCTCAAGCCACGCCT
e2 AK125728	RV	TTCATCCACACTTCTCAGG
	FW	CTACTATGGCGCTGTGTAA
e3 AK125728	RV	TCGGTGGCTTTGTGCATCATTT
	FW	TCCCTGCTGAAATACCTTAGTG
e4 AK125728	RV	AGAGGCCAGTGGGAATATGA
	FW	CACAATGAGCATTTACCCAGC
e5 AK125728	RV	ATTTGCCAGTGAGTTGAACAG
	FW	CTGCCTTTCAAGGAAAGTAAC
e6 AI138360	RV	CAATGGGTGGATATTTACTGC
	FW	TGCATCCCTAAATTCATCCCT
CYR61 mRNA (e4)	RV	CACAAATCCGGGTTTCTTTCAC
	FW	AAACAACTTCATGGTCCCAGTG
ZNHIT6 mRNA (e10)	RV	AAGTACTATGCTGATCAAGTGC
	FW	TGCAGCAGACTCTGCATTGAT
ZNHIT6 mRNA (e4-e5)	RV	GAATTCTCCTTCCTCTTGGTG
	FW	GGCAAGAACAGCGGACCATA
SYDE2 mRNA (e3)	RV	TGTAAGTTCAGGTGAGTCTCC

	FW	CCGATACCATCTTGATACCAG
C1orf52 mRNA (e3)	RV	CTTCTGGTAGAAGCCTAGCTT
	FW	TGAGACCTACACCACTGAGAA
BCL10 mRNA (e3)	RV	AGGGCGTCGTGCTGGATTCT
	FW	CCAGATGGAGCCACGAACAA
BCL10 mRNA (e3-2)	RV	CAGGTCTGGGAAGTGTAGTTG
	FW	CCAGATGGAGCCACGAACAA
p15 mRNA (e2)	RV	AATATCCCTGGAAATCCGCTTC
	FW	TTTCTTACCCAATTTCCCACCC
p16 mRNA (e1-e2)	RV	ACCACCAGCGTGTCCAGGAA
	FW	CTGCCCAACGCACCGAATAG
p16/p14^{ARF} (e2)	RV	GACCTTCCGCGGCATCTATG
	FW	CGATGTCGCACGGTACCTG
p14^{ARF} mRNA (e1)	RV	GCTGGCTCCTCAGTAGCATC
	FW	TTGGTGACCCTCCGGATTCG
GAPDH mRNA	RV	CTCTTCCTCTTGTGCTCTTGC
	FW	TGACAACGAATTTGGCTACAGC
U6 snRNA[5]	RV	AACGCTTCACGAATTTGCGT
	FW	CTCGCTTCGGCAGCACA
pre-rRNA 45S[6]	RV	GCGTCTCGTCTCGTCTCACT
	FW	GAACGGTGGTGTGTCGTTC
HMGA1 (e3a-e4)	RV	TTAGGTGTTGGCACTTCGCTG
	FW	ATGAGTGAGTCGAGCTCGAAG
HMGA2 (e3-e6)	RV	ATCCAACTGCTGCTGAGGTAG
	FW	AAGCCACTGGAGAAAAACGGC
PRKCA1 (i1)	RV	ACAGCTGACCTCCTCTTAACC
	FW	AGCCACCTCTATGAGTGATTTC
SND1 (i10)	RV	AGTCATACACCAACCTATCTGC
	FW	GTATTCACTGGGGTCACCTAC
H2A.Z (e3-e4)	RV	CGAGGGGTAATACGCTTTACC
	FW	GGGCCGTATTCATCGACACC
-235 nt SYDE2	RV	GTCGGCGTCGTAGTCGTCT
	FW	AGAGCCATGGGCTCGCTCA
+136 nt VAD	RV	CAGGCGTGGCTTGAGGAAAC
	FW	TGTCTCTGCGGCAGCCTATC
-300 nt DDAH1-S	RV	AAGCAGCGAGGGCAAGAAGT
	FW	ACCTGCGACAGAACACAGGTA
-1Kb up CYR61	RV	TTCCTCACGGATGCAGGAGA
	FW	CTTGCGGTCTTTGCAGGTGA
CYR61 (e1)	RV	TGGTCAAGTGGAGAAGGGTGA
	FW	CGCTGCACACCAGCTTGTTG
CYR61 (e3)	RV	AACTTTGACCAGCCGAGGGTT
	FW	CAGACCCTGTGAATATAACTCC
-100 nt ZNHIT6	RV	GGAATAGCCTGCTTGACGCG
	FW	TTCGCCCAGAGAATCCGGCA
p15 (e1)	RV	GGCCGTAAACTTAACGACACT
	FW	GGGAGGGTAATGAAGCTGAG
p16 (prom, -915 nt)[7]	RV	AAGCCTTAAGAACAGTGCC
	FW	CTCAAAGCGGATAATTCAA
GAPDH (e1)	RV	GCGACGCAAAAGAAGATGCG
	FW	AAATTGAGCCCGCAGCCTCC

Supplementary References

1. Ge, X., Rubinstein, W. S., Jung, Y. C. & Wu, Q. Genome-wide analysis of antisense transcription with Affymetrix exon array. BMC Genomics 9, 27 (2008).

2. Iacovoni, J. S. et al. High-resolution profiling of gammaH2AX around DNA double strand breaks in the mammalian genome. EMBO J 29, 1446-57 (2010).

3. Jeanblanc, M. et al. Parallel pathways in RAF-induced senescence and conditions for its reversion. *Oncogene* **31**, 3072-85 (2012).

4. Narita, M. et al. A novel role for high-mobility group a proteins in cellular senescence and heterochromatin formation. *Cell* **126**, 503-14 (2006).

5. Galiveti, C.R., Rozhdestvensky, T.S., Brosius, J., Lehrach, H. & Konthur, Z. Application of housekeeping npcRNAs for quantitative expression analysis of human transcriptome by real-time PCR. *Rna* **16**, 450-61 (2010).

6. Murayama, A. et al. Epigenetic control of rDNA loci in response to intracellular energy status. *Cell* **133**, 627-39 (2008)

7. Bracken, A.P. et al. The Polycomb group proteins bind throughout the INK4A-ARF locus and are disassociated in senescent cells. *Genes Dev* **21**, 525-30 (2007).
